# Cancer Associated Fibroblasts: The Architects of Stroma Remodeling

**DOI:** 10.1002/pmic.201700167

**Published:** 2018-02-01

**Authors:** Alice Santi, Fernanda G. Kugeratski, Sara Zanivan

**Affiliations:** ^1^ Cancer Research UK Beatson Institute Glasgow UK; ^2^ Institute of Cancer Sciences University of Glasgow Glasgow UK

**Keywords:** blood vessel, cancer, cancer associated fibroblasts, endothelial cell, extracellular matrix, immune system, invasion, proteome, secretome, tumor microenvironment

## Abstract

Fibroblasts have exceptional phenotypic plasticity and capability to secrete vast amount of soluble factors, extracellular matrix components and extracellular vesicles. While in physiological conditions this makes fibroblasts master regulators of tissue homeostasis and healing of injured tissues, in solid tumors cancer associated fibroblasts (CAFs) co‐evolve with the disease, and alter the biochemical and physical structure of the tumor microenvironment, as well as the behavior of the surrounding stromal and cancer cells. Thus CAFs are fundamental regulators of tumor progression and influence response to therapeutic treatments. Increasing efforts are devoted to better understand the biology of CAFs to bring insights to develop complementary strategies to target this cell type in cancer. Here we highlight components of the tumor microenvironment that play key roles in cancer progression and invasion, and provide an extensive overview of past and emerging understanding of CAF biology as well as the contribution that MS‐based proteomics has made to this field.

## 1 The Tumor Microenvironment

**Figure 1:**
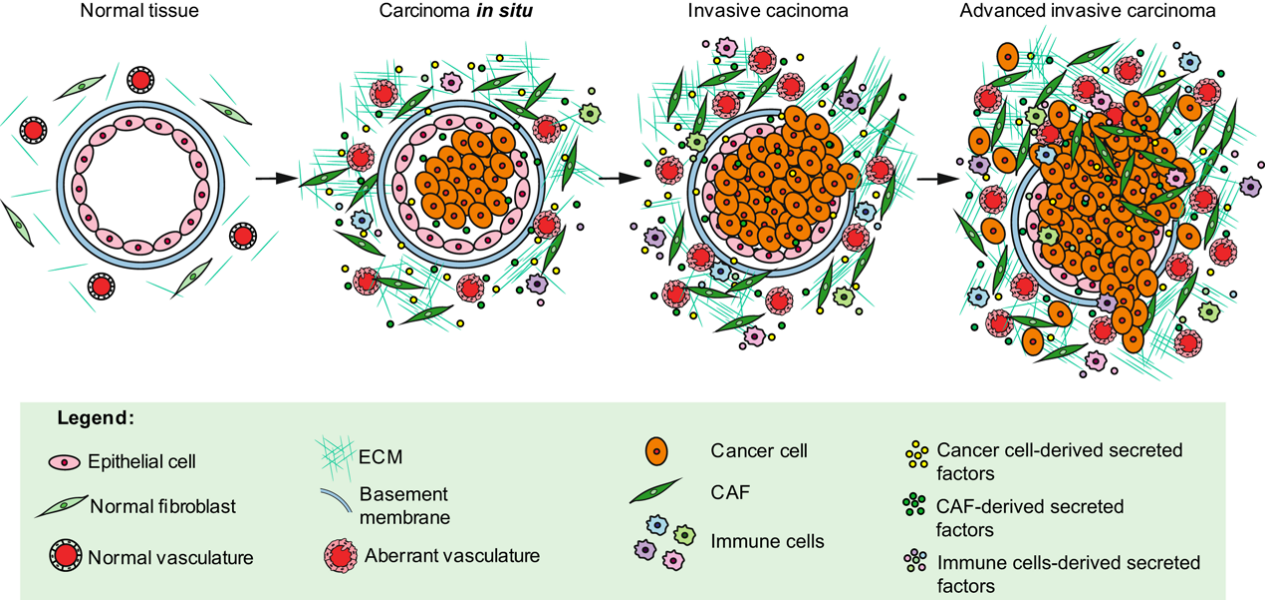
Tumor progression. Schematic representation of tumor development with highlighted stromal components that contribute to progression and invasion.

Neoplastic lesions in situ are confined within a layer of basement membrane which physically separates epithelial cells from the underlying stromal compartment. During malignant transformation, cancer cells acquire invasive properties, breach the basement membrane, and invade the surrounding stroma (referred to also as tumor microenvironment) (Figure 1). In tumors in situ, cancer and stromal cells can communicate through the basement membrane; in invasive tumors, cancer and stromal cells are in direct contact and establish a complex crosstalk that evolves alongside tumor development and leads to alterations of the microenvironment^1^, ^2^ In healthy tissue the stroma is populated by few resident fibroblasts embedded within a vascularised physiological extracellular matrix (ECM). In tumors, the stroma contains increased number of fibroblasts, which are pathologically activated and called cancer associated fibroblasts (CAFs), blood/lymphatic vessels, which are often dysfunctional, immune cell infiltrates, and an ECM profoundly remodeled compared with the physiological one. CAFs are a heterogeneous and highly secretory population of cells which play key roles in constructing a microenvironment that modulates functions and behavior of the surrounding cells during tumor progression. For this reason CAFs have been referred to as the “architects of cancer pathogenesis”^3^ and are emerging as a promising therapeutic target.

We will briefly describe three major components of the tumor stroma—blood/lymphatic vessels, immune system, and ECM—and provide a detailed overview of CAFs and how they regulate those stromal compartments.

### 1.1 The Tumor Vasculature

The tumor vasculature has fundamental roles in cancer because it fuels the uncontrolled growth of cancer cells by refurbishing the tumor with nutrients and oxygen and removing waste products. Furthermore, it provides a route for the cancer cells to escape the primary tumor to form distant metastases.^4^, ^5^ Endothelial cells (ECs) are the key players in the formation and functional regulation of vessels. ECs line the blood vessel wall and are in direct contact with the blood, pericytes, or smooth muscle cells which wrap around the ECs, and macrophages which help the remodeling of the vasculature.^2^, ^6^, ^7^

Tumor vessels originate via different processes, including growth from preexisting vessels that infiltrate the tumor (angiogenesis), de novo formation from EC precursors recruited within the tumor (vasculogenesis), or hijacking of the existent vasculature by the cancer cells (co‐option).^8^, ^9^, ^10^, ^11^ Tumor vascularization is driven by numerous factors, including vascular endothelial growth factor A (VEGFA), stromal cell‐derived factor 1 (also known as C‐X‐C motif chemokine 12, CXCL12), fibroblast growth factors (FGFs), platelet‐derived growth factor alpha (PDGFα), transforming growth factor beta (TGF‐β), angiopoietin 2 (ANG‐2), interleukin 8 (IL‐8), IL‐6, matrix metalloproteinase‐2 (MMP2), MMP9 (reviewed in 12, 13, 14). Due to the excessive secretion of those factors by cancer and stromal cells, the organization and structure of the tumor vasculature is often abnormal. Vessels have irregular branches and are tortuous. Moreover, they are structurally unstable due to defective coverage with pericytes and basement membrane, and unstable EC–EC and EC–pericyte intercellular junctions.^15^, ^16^, ^17^, ^18^, ^19^ Tumor dysplasia and the rapid growth of the tumor mass can create high density regions where new vessels cannot penetrate and the resident ones are compressed.^20^ These abnormalities lead to the establishment of a tumor vasculature that is leaky. This aids cancer cell intravasation to form distant metastases,^21^ impairs immune cell infiltration,^22^ and creates hypoxic and necrotic tumor regions.^23^ Dysfunctional vessels also hamper the transport and efficacy of chemo and radiation therapies.^12^

Also lymphatic vessels provide a route for metastatic spread. In physiological conditions, the lymphatic vasculature controls fluid homeostasis, lipid absorption, and immune surveillance. In tumors, lymphatic vessels undergo a structural remodeling through lymphangiogenesis and enlargement of the surface area. These changes enhance lymphatic EC–cancer cell contact and facilitate the access of cancer cells into the lymphatic system to establish lymph node metastases.^24^, ^25^, ^26^, ^27^ The main drivers of lymphangiogenesis are VEGFC and VEGFD, which can be secreted by cancer cells, CAFs, and immune cells.^28^, ^29^, ^30^

### 1.2 The Immune System

Intratumoral immune infiltrates are composed of various cell types of the innate (macrophages, dendritic cells, mast cells, granulocytes, natural killer cells) and adaptive immune system (B cells and CD4^+^ and CD8^+^ T lymphocytes, natural killer T cells, and γδ T cells), and their composition is determined by tumor‐derived signals, such as the expression of cytokines, and the expression of genes specific of the normal tissue of origin.^31^, ^32^ As an example, there are similar numbers of macrophages in the stroma of human lung adenocarcinoma and normal lung, but only macrophages within the tumor lesion have an immunosuppressive and pro‐tumorigenic phenotype.^33^ The main role of immune infiltrates is to recognize and eliminate cancer cells. However, their tumor blocking functions can be suppressed by cytokines and growth factors secreted by intratumoral myeloid, cancer, and stromal cells. A key example is the tumor‐induced expression and activation of receptors on T cells, such as programmed cell death protein‐1 (PD1), T‐cell immunoglobulin mucin 3 (Tim‐3), and cytotoxic T‐lymphocyte protein 4 (CTLA4), which inhibit their antitumorigenic functions.^34^, ^35^ Another mechanism that inhibits immune infiltrate involves ECs. Tumor ECs upregulate adhesion molecules, such as stabilin‐1 (STAB1, also known as CLEVER1) mucosal vascular addressin cell adhesion molecule 1 (MADCAM1), and activated leukocyte cell adhesion molecule (CD166), that selectively promote the infiltration of regulatory T cells, which secrete immunosuppressive cytokines.^36^, ^37^

### 1.3 The Extracellular Matrix

The ECM is a complex network of macromolecules whose structure and composition define its biochemical and biomechanical properties. Collagens, laminins, glycoproteins such as tenascin C (TNC) and fibronectin, proteoglycans, and polysaccharides are major ECM components.^38^, ^39^ The ECM provides structural support to the cells and transduces biomechanical signals that modulate cellular functions, including motility, proliferation, and differentiation. Moreover, it binds to growth, survival, motility, and angiogenic factors such as TGF‐β, FGF, PDGF, epidermal growth factor (EGF), hepatocyte growth factor (HGF), and VEGF, thus being a reservoir of factors whose availability depends on ECM remodeling.^40^, ^41^

Tumor progression is often accompanied by a desmoplastic reaction, such that the ECM can constitute the majority of the tumor mass. Both cancer and stromal cells contribute to deposition of ECM, and its properties alter tumor features, such as the potential to form metastasis.^42^ Cells modify the ECM not only by secreting ECM components, but also by secreting enzymes which modify the ECM, such as transglutaminases (TGMs) and lysyl oxidases (LOXs), which crosslink ECM components.^43^, ^44^ Conversely, MMPs, a disintegrin and metalloproteinases (ADAMs), and a disintegrin and metalloproteinase with thrombospondin motifs (ADAMTSs) proteins proteolytically degrade ECM components and release ECM‐bound soluble factors.^45^, ^46^, ^47^ The activity of these proteases is inhibited by tissue inhibitors of metalloproteinases.^48^ The dysregulated activity of these enzymes, together with the excessive deposition of ECM components and their reduced turnover, are typical in malignant lesions. Thus tumor ECM is different from physiological ECM and directly influences tumor progression (reviewed in 49, 50). For example, the tumor ECM is usually stiffer than physiological ECM, and this plays key roles in maintaining CAF phenotype, enhancing cancer and stromal cell invasion, cancer cell‐endothelium interactions, epithelial to mesenchymal transition (EMT) transformation, and immune cell recruitment.^38^, ^51^, ^52^, ^53^, ^54^

### 1.4 CAFs

#### 1.4.1 The Origins of CAFs

In physiological conditions, fibroblasts are low proliferative spindle‐shaped cells located in the connective tissue of most organs. Fibroblasts secrete ECM components and ECM‐remodeling enzymes to maintain the homeostasis of the stroma and define the structural integrity and mechanical properties of organs. Fibroblasts also control the polarity and function of the epithelium by producing basement membrane.^55^, ^56^ In 1971, Giulio Gabbiani described for the first time the existence of fibroblastic cells with contractile properties, namely myofibroblasts, in the granulation tissue, and hypothesized that myofibroblasts had reparative activity during wound healing.^57^ Later studies revealed that the contractility was primarily mediated by the expression of ED‐A,^58^ a splice variant of cellular fibronectin, and the actin isoform alpha‐smooth muscle actin (α‐SMA). The mechanical stress generated after a wound and the inflammatory factors released by injured tissues induce expression of ED‐A and TGF‐β1 in normal resident fibroblasts. TGF‐β1, in turn, induces the expression of α‐SMA, which is incorporated in the stress fibers. α‐SMA was initially described in smooth muscle cells, but is now a widely used marker of myofibroblasts and myofibroblast‐like cells.^59^ In 1979, cells with morphological and molecular properties of myofibroblasts were described in the stroma of solid tumors.^60^ These myofibroblastic cells are referred to as CAFs.

CAFs originate from the activation of resident fibroblasts^61^ or other precursor cells. CAFs can derive from bone marrow‐derived mesenchymal stem cells,^62^ epithelial cells,^63^, ^64^ carcinoma cells,^63^ ECs,^65^ pericytes,^66^ smooth muscle cells,^63^ adipocytes,^67^ fibrocytes,^68^ or from some specialized cells such as stellate cells in pancreas and liver,^69^ myoepithelial cells in breast,^70^ and pericryptal myofibroblasts in the gastrointestinal tract.^71^ This spectrum of precursors explains, at least in part, the heterogeneity of CAFs. CAF activation is triggered by a variety of stimuli, including cancer cell‐derived TGF‐β1, PDGFα, PDGFβ, basic FGF (bFGF, also known as FGF2), and IL‐6,^72^, ^73^, ^74^, ^75^, ^76^ and environmental stimuli, such as hypoxia, oxidative stress, and matrix stiffness.^52^, ^77^, ^78^ All these stimuli may cooperate to determine different CAF phenotypes further contributing to their heterogeneity. As a consequence, while α‐SMA represents the oldest and commonly used marker to assess CAF phenotype, not all CAFs express α‐SMA. Prolyl endopeptidase FAP (also known as fibroblast activation protein), fibroblast‐specific protein 1 (FSP1, also known as S100A4), and the mesenchymal cell marker vimentin are expressed at higher levels in CAFs compared to non‐activated fibroblasts and are also considered CAF markers. Conversely, the cell surface protein CD36 and caveolin 1 (CAV1) levels decrease upon activation (CAF markers reviewed in 13, 79). None of these markers are exclusively expressed in CAFs, and the majority are not synchronously expressed. For example, a distinct CAF subpopulation FSP1^+^, α‐SMA^−^, PDGFRβ^−^, and NG2^−^ has been identified within the stroma of tumors isolated from Rip1Tag2 model of β‐cell carcinogenesis and murine orthotopic 4T1 breast tumors.^80^ A CAF subpopulation α‐SMA^high^, which localizes adjacent to the cancer cells, and one α‐SMA^low^ and IL‐6^high^, which secretes inflammatory factors and is located distant from the cancer cells, have been characterized in murine model of pancreatic adenocarcinoma (PDAC).^81^ Several combinations of α‐SMA^+^, S100A4^+^, and FAP^+^ CAF subpopulations have been identified in MMTV‐PyMT breast tumors isolated at different stages of the progression, and in human cervical, head and neck and vulval squamous cell carcinoma (SCC) CAF lines.^52^ In tissue sections of esophageal carcinoma, colorectal adenocarcinoma, and head and neck SCC, a subset of α‐SMA^+^ CAFs was found associated with regions with elongated collagen fibers.^82^ While CAF heterogeneity starts to become well documented, future studies need to address the extent of this heterogeneity during tumor progression, in different tumor types, and whether distinct functions are associated to each CAF phenotype.

**Figure 2:**
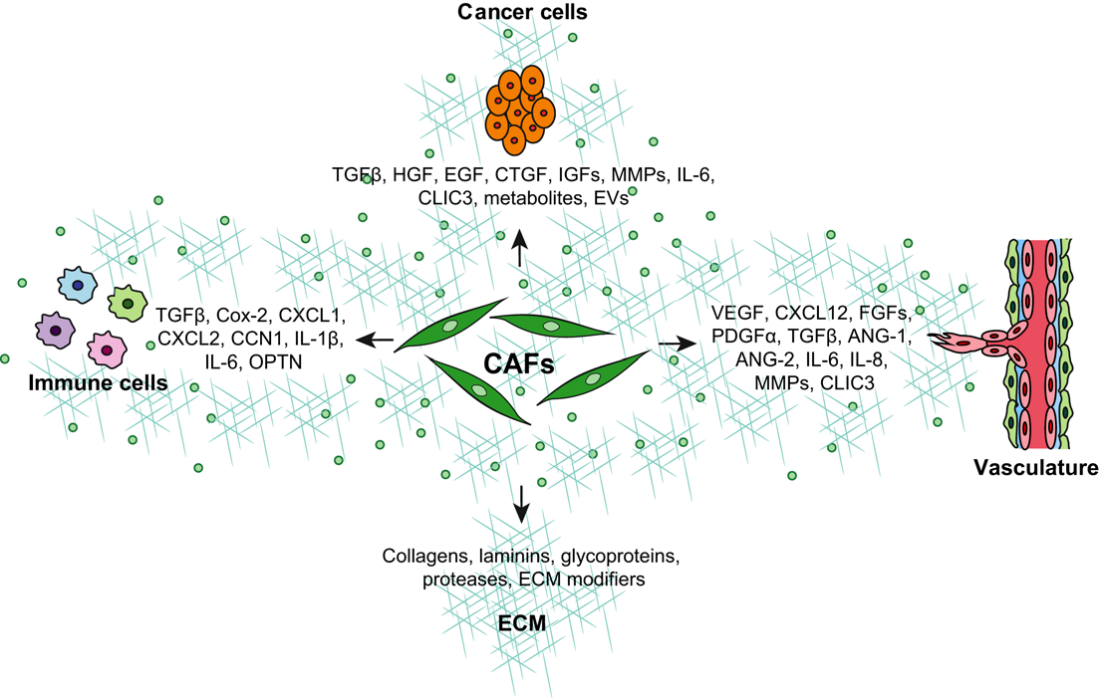
CAF functions. Schematic representation of key CAF functions and CAF‐derived factors involved.

Upon activation, CAFs secrete a vast repertoire of growth factors, such as HGF, EGF, connective tissue growth factor (CTGF) and insulin‐like growth factor (IGF), cytokines, including CXCL12 and IL‐6, extracellular vesicles (EVs), metabolites, ECM components, particularly collagens, fibronectin and TNC, and ECM‐remodeling enzymes, such as MMPs, LOXs, and TGMs. All these factors directly affect the behavior of the surrounding cells and remodel the ECM (see below and Figure 2). Thus CAFs aid tumor development, from the early stages of tumorigenesis until cancer cells colonize distant organs to form metastasis.^3^, ^13^, ^63^ CAFs also contribute to resistance to therapy.^83^, ^84^

#### 1.4.2 CAFs Promote Cancer Cell Migration and Invasion

CAFs modulate migratory and invasive traits of cancer cells indirectly by remodeling the ECM and directly by influencing cancer cell phenotype and aiding cancer cell migration.

In organotypic co‐cultures of head and neck SCC CAFs with SCC12 cancer cells, CAFs deposit ECM tracks that lead the collective invasion of cancer cells in a mechanism dependent on protease‐ and force‐mediated ECM remodeling. Blocking α3 and α5 integrin or Rho associated protein kinase (ROCK) in CAFs decreased their ability to produce ECM tracks to facilitate cancer cell migration.^85^ Accordingly, in patient‐ and PyMT‐derived mammary CAFs, ROCK was required for the expression of cytoskeletal regulators and stiffening of the ECM via nuclear localization of the transcriptional coactivator YAP (YAP1). Silencing YAP1 in PyMT CAFs reduced their ability to contract 3D collagen gels and to assemble stiff ECM, and this reduced the invasiveness of SCC12 cancer cells in organotypic co‐cultures.^52^ In CAFs, ROCK‐dependent actomyosin contractility was also induced through GP130‐IL6ST/JAK1 signaling activated by oncostatin M and blocking JAK1 signaling in CAFs reduced their capability to contract gel and induce SCC12 invasion.^86^ Highlighting the importance of ROCK signaling in CAFs and its potential as target for therapies, in murine models of PDAC targeting ROCK with small molecules reduced fibroblast/stellate cell activation, ECM deposition and this, in turn, enhanced gemcitabine uptake and improved survival.^87^, ^88^

Also micro RNAs control CAF contractility. The miR‐200s negatively regulate the ability of human mammary CAFs to contract collagen gels and promote cancer cell invasion. MiR‐200s‐silenced normal fibroblasts acquired features of CAFs, such as increased levels of α‐SMA and accelerated migration and invasion, in vitro. Conversely, ectopic expression of miR‐200s in CAFs reduced α‐SMA levels, cell invasion, and the ability to contract collagen gels. Subcutaneously cotransplanted CAFs overexpressing miR‐200s with MDA‐MB‐231 breast cancer cells formed smaller and less invasive tumors compared with those formed with control CAFs.^89^

The ability of CAFs to remodel the ECM is controlled also by intratumoral hypoxia,^90^ a typical hallmark of solid tumors. Hypoxia induces secretion of prolyl 4‐hydroxylase subunit alpha‐1 and alpha‐2 (P4HA1, P4HA2), which are required for collagen deposition, and procollagen‐lysine,2‐oxoglutarate 5‐dioxygenase 2 (PLOD2), which is essential for the alignment of the ECM fibers. Thus, the ECM deposited by hypoxic fibroblasts promoted changes in morphology, adhesion, and migration of breast cancer cells and enhanced invasion.^91^ Moreover fibroblasts expressing activated hypoxia‐inducible factor 1 alpha (HIF1α) were pro‐tumorigenic and pro‐metastatic when cotransplanted in MDA‐MB‐231 xenografts.^92^ Conversely, hypoxia decreased expression of α‐SMA and impaired contractile forces and the ability to remodel the ECM in vulval and head and neck SCC CAFs cultured in 3D collagen gels. In vivo, genetic or pharmacological ablation of prolyl hydroxylase 2 (EGLN1 also known as PHD2), which stabilizes the transcription factor HIF1α, in CAFs cotransplanted orthotopically with 4T1 breast cancer cells decreased tumor stiffness, invasion and liver metastasis.^93^ Accordingly, HIF1α‐depletion,^94^ but not depletion of PHD2,^95^ in fibroblasts before tumor onset accelerates tumorigenesis in a MMTV‐PyMT model. It is clear that how hypoxia influences CAF phenotype and their pro‐invasive functions is still controversial. Future studies are required to address whether hypoxia has distinct effects on different CAF subpopulations or whether the effect depends on tumor type or other tumor or stromal factors.

Cancer cells that undergo EMT have enhanced migratory and invasive traits while losing adhesive properties. CAFs play a role in this process by promoting EMT. Patient‐derived CAFs isolated from prostate tumors triggered EMT and invasiveness of PC3 prostate cancer cells through a metalloproteinase‐dependent mechanism. As a result CAFs cotransplanted with PC3 cancer cells showed enhanced metastasis.^73^ The conditioned medium (CM) of cultured mammary CAFs was able to induce EMT and invasive behavior of MCF7 breast cancer cells and MCF10A breast epithelial cells.^96^ In line with this report, treatment of MCF7, T47D, and MDA‐MB‐231 cells with CM from patient‐derived mammary CAFs promoted the expression of EMT markers through TGF‐β signaling activation.^97^ CAFs isolated from non‐small cell lung cancer (NSCLC) tissue expressed more IL‐6 compared to normal lung fibroblasts, and their CM induced an IL‐6‐dependent EMT in NSCLC cells and resistance to cisplatin treatment. Moreover, IHC analysis of NSCLC tissues showed that stromal IL‐6 levels correlate with EMT phenotype of cancer cells.^98^ CAFs isolated from human gastric carcinoma tissue secrete Galectin 1 (LGALS1), which binds to β1 integrin in MGC‐803 gastric cancer cells thus inducing EMT via upregulation of the glioma‐associated oncogene 1 (GLI1).^99^ Finally, CAFs can support invasion by establishing physical interactions with the cancer cells. Human lung adenocarcinoma and vulval SSC CAFs promoted A431 SCC cancer cell migration and invasion by establishing heterophilic N‐cadherin/E‐cadherin interactions.^100^ Moreover, CAFs can co‐travel in the blood with circulating murine metastatic lung carcinoma cancer cells to support cancer cell viability and provide them with a growth advantage at the metastatic site.^101^ A better understanding of the signaling downstream of such heterotypic cell–cell interactions could provide insights to develop strategies to target invasion and metastasis.

#### 1.4.3 CAFs Restrain Tumor Progression

CAFs are mostly described as pro‐tumorigenic. However, CAFs can also restrain tumorigenesis. Depletion of α‐SMA^+^ proliferating cells either at non‐invasive pancreatic intraepithelial neoplasia or PDAC stage in *KRas^G12D^* model accelerated the progression of the disease.^102^ Similarly, deletion of the desmoplastic driver Sonic hedgehog (Shh) in the cancer cells of a PDAC model promoted aggressiveness.^103^ Patient‐derived mammary fibroblasts, 199Ct CAF line, and WI38 and HS68 fibroblast lines were found to secrete high levels of slit homolog 2 protein (SLIT2). CAF‐derived SLIT2 suppresses invasion of breast cancer cells expressing its receptor roundabout homolog 1 (ROBO1), through inhibition of PI3K and β‐catenin signaling. Consistently, tumor growth was suppressed in ROBO1 expressing HCC1937 xenografts, while enhanced in ROBO1‐depleted MDA‐MB‐231 xenografts cotransplanted with SLIT2 expressing 199Ct fibroblasts. Finally, in breast cancer patients, high SLIT2 levels in the stroma and high ROBO1 levels in the cancer cells were indicative of better clinical outcome.^104^ Triple negative breast cancer (TNBC) cells suppressed the expression of the secreted protein asporin in stromal fibroblasts. Notably, CAF‐derived asporin inhibits TGF‐β signaling and EMT in breast cancer cells, and overexpression of asporin in patient‐derived normal mammary fibroblasts cotransplanted with MDA‐MB‐468 xenograft reduced tumor growth and metastasis.^105^

The conundrum that CAFs can either promote or restrain tumors needs further attention in the future. More effort should be devoted to identify the molecular mechanisms behind these opposite functions to provide insights for the development of therapies that specifically target pro‐invasive CAF functions, while promoting those that restrain tumors.

#### 1.4.4 CAFs Control Vascular and Immune Systems

Early evidence that CAFs induce tumor vascularization came from transgenic mice expressing GFP under the VEGF promoter and transplanted with mammary carcinoma MCaIV or hepatocellular carcinoma HCaI cells, or crossed with PyMT mice. In these models cancer cells induced VEGF expression in intra‐ or peri‐tumoral fibroblasts and these VEGF‐expressing regions were vascularized.^106^ The role of CAFs in tumor vascularization has been confirmed in other models. In an HPV cervical carcinogenesis model, PDGFRs‐expressing CAFs induced cancer cell proliferation and angiogenesis. The pro‐angiogenic effect was due to FGF2 secretion, which was induced by cancer cell‐derived PDGF, and this was abrogated by pharmacological treatment to block stromal PDGFR signaling.^107^ After co‐transplantation of patient‐derived mammary CAFs and MCF7 breast carcinoma cells expressing activated HRas oncogene (MCF7‐HRas), CAFs induced the formation of tumors extensively vascularized compared with those grown with normal fibroblasts. In this model, CAFs secreted high levels of CXCL12 which induced endothelial progenitor cell recruitment.^108^ Moreover, PyMT CAFs embedded in matrigel plugs subcutaneously transplanted induced vascularization of the plug, and silencing of YAP1 decreased this pro‐angiogenic capacity.^52^ Stromal deletion of the tumor suppressor *Pten* promoted angiogenesis and tumorigenesis in MMTV‐*ErbB2/neu* and MMTV‐PyMT breast cancer models. In MMTV‐*ErbB2/neu* tumors, stromal *Pten* depletion before tumor onset induces Ets2‐dependent expression of ECM remodeling, wound healing, and chronic inflammation factors in CAFs, as well as intratumoral recruitment of macrophages. Stromal *Ets2* depletion in stromal *Pten*‐null mice reduces growth, vascularization, and macrophage recruitment in tumors grown from orthotopically transplanted NT2.5 *ErbB2*‐expressing cancer cells.^109^ The pool of CAF‐secreted cytokines also contributes to recruit and modulate polarization and functions of immune cells. The expression of pro‐inflammatory factors, such as cyclooxygenase 2 (COX2), CXCL1, CXCL2, the protein CYR61 (CYR61/CCN1), IL‐1β, IL‐6, and osteopontin, is mediated by NF‐kB in dysplasia‐derived CAFs from a murine model of HPV skin carcinogenesis; CAFs required NF‐kB to promote tumor growth, macrophage recruitment, and vascularization when orthotopically cotransplanted with murine PDSC5 skin carcinoma cells.^110^ IL‐6 secreted by normal skin fibroblasts co‐cultured in physical contact with monocytes promoted monocyte differentiation into macrophages.^111^ Accordingly, in an orthotopic 4T1 model, FAP^+^ CAF‐deleted tumors had reduced levels of IL‐6 and IL‐4 while increased IL‐2 and IL‐7, and this enhanced the recruitment of antitumor immune cells (DCs and CD8^+^ T cells), while inhibiting the recruitment of pro‐tumorigenic ones (F4/80^+^ tumor associated macrophages, TAMs, CD11b^+^/Gr‐1^+^, myeloid derived suppressor cells, MDSCs, and CD4^+^/FOXP3^+^ T regulatory cells, Tregs). As a consequence, FAP^+^ CAF depletion inhibited the formation of lung metastasis and enhanced the anti‐metastatic effects of chemotherapy (doxorubicin).^112^ In the KPC murine model of PDAC, FAP^+^ CAFs had pro‐inflammatory and desmoplastic signatures and suppressed cancer immune surveillance. Partial toxin‐induced depletion of FAP^+^ CAFs, as well as pharmacological blockage of CXCL12 signaling by inhibiting its receptor C‐X‐C chemokine receptor 4 (CXCR4), reduced tumor growth and enhanced T cell accumulation within the tumor, thus sensitizing tumors to α‐PDL1 treatment. This effect was possibly due to the capability of CXCL12 to block infiltration of CXCR4‐expressing T cells.^113^ MRC5 fibroblasts, foreskin fibroblasts, and mammary CAFs co‐cultured with MDA‐MB‐231, Hs578T, MDA‐MB‐436, MDA‐MB‐157, or HCC1937 basal‐like ER^−^ breast cancer cells induced interferon‐stimulated genes, which drove mechanisms of resistance to chemotherapy and radiation through the activation of STAT1 in the cancer cells. STAT1 activation was induced through the pattern recognition receptor RIG‐I, which was activated by binding to RNA, 7SL, Cytoplasmic 1 (RN7SL1). RN7SL1 was transferred to the cancer cells through fibroblast‐derived EVs. Activation of RIG‐I in the cancer cells led to NOTCH/MYC pathway activation in fibroblasts which, in turn, enhanced stromal RN7SL1 availability, thus establishing a positive feed‐back loop. RN7SL1‐containing EVs from MYC‐activated mouse embryonic fibroblasts inoculated into mice were able to activate circulating myeloid and dendritic cells, suggesting that fibroblast‐derived RNAs can control tumor progression by horizontal transfer to cancer cells and also circulating inflammatory cells.^114^, ^115^

## 2 CAFs and MS‐Proteomics

Modern MS‐based proteomics allows the measurement of cell proteome and secretome, such as CM and ECM from cell cultures and ECM from tissue samples, at global scale. Fibroblasts are highly secretory cells, thus MS‐proteomics provides unique opportunities to understand CAF biology and their ability to alter the tumor microenvironment.

### 2.1 CAF Signatures Unraveled by Total Proteome Analysis

Several proteomic studies have assessed differences between CAFs and their normal fibroblast counterpart to identify potential markers and regulators of CAF functions.

Comparative proteomic analysis of murine CAFs isolated from sporadic colon cancer with their matched normal fibroblasts revealed upregulation of latent transforming growth factor beta binding protein 2 (LTBP2), farnesyl diphosphate synthase (FDPS), and cadherin 11 (CDH11) in CAFs. LTBP2 and CDH11 expression were both induced by TGF‐β and elevated in the stroma of colon cancer tissues.^116^ α‐SMA^+^ and vimentin^+^ cultured fibroblasts derived from 11 pairs of human gastric cancer and tumor‐adjacent tissues expressed different levels of proteins related to actin filaments, and this mirrored higher migration and proliferation rates of CAFs.^117^ Similarly, the cellular proteome of α‐SMA^+^ CAFs derived from two human invasive mammary ductal carcinoma and SMA^−^ normal fibroblasts from matched non‐malignant adjacent region were different. Notably, tumor‐adjacent fibroblasts had an intermediate proteomic profile between normal fibroblasts and CAFs.^118^ Proteome and secretome analysis of immortalized fibroblasts isolated from normal breast and MCF7‐HRas cell‐activated CAFs^119^ defined that CAFs had a myofibroblastic phenotype. Moreover, it unraveled altered levels of proteins related to metabolism, cell morphogenesis, differentiation, adhesion, and motility. Consistent with this molecular portrait, CAFs induced invasive behavior of A2780, MCF10DCIS.com, and MDA‐MB‐231 cancer cells and human umbilical vein endothelial cells.^120^

Some studies, instead, compared the whole proteome of patient tumor tissues and neighboring regions. To discriminate between cancer cell and stromal contribution to the proteomic data, cell‐type specific proteome profiles were used as reference. For breast cancer, human ZR‐75‐1 cancer cells and TGF‐β‐stimulated or IL‐1β‐stimulated human fibroblasts to mimic myofibroblast‐like or inflammation‐induced CAFs, respectively, were used. Fibroblast‐derived proteins were hardly detected in biopsies from central tumor regions, while they were in those from the surrounding tissue. Most of the identified proteins, including fibulin‐5 (FBLN5), solute carrier family 2 facilitated glucose transporter member 1 (SLC2A1) and cell surface glycoprotein MUC18, were more abundant in TGF‐β‐induced fibroblasts, indicating the presence of myofibroblast‐like CAFs in peripheral tumor regions.^121^ For hepatocellular carcinomas, reference profiles were generated from cultured patient‐derived cancer cells and CAFs. Higher levels of PLOD2, eukaryotic translation initiation factor 3 subunit H (EIF3H), ubiquitin carboxyl‐terminal hydrolase isozyme L1 (UCHL1), P4HA1, actin‐related protein 2 (ACTR2), protein S100A13, mitochondrial oligoribonuclease (REXO2), and the chloride intracellular channel 4 (CLIC4) were measured in the tumor. These proteins were more abundant in CAFs than cancer cells, suggesting that CAFs contribute to a large extent to the proteome of hepatocellular carcinomas.^122^

### 2.2 CAF Metabolism Unraveled by Total Proteome Analysis

MS‐proteomics contributed to uncover metabolic rewiring associated with fibroblast activation. Notably, highly metabolically active cancer cells release reactive oxygen species, which, in turn, induce oxidative stress in the adjacent fibroblasts and activate HIF1, through the stabilization of HIF1α subunit, and NF‐kB. HIF1 and NF‐kB activation increases autophagy, which mediates CAV1 degradation.^77^, ^123^, ^124^, ^125^, ^126^ MS analysis provided first hints that HIF1α and NF‐kB activation, as well as CAV1 downregulation, are key drivers of the metabolic rewiring that occurs in fibroblasts upon activation into CAFs. Overexpression of HIF1α or the NF‐kB‐activator kinase IKBKE induced upregulation of glycolytic enzymes in hTERT‐BJ1 human immortalized foreskin fibroblasts.^92^ Similarly, CAV1‐depleted murine embryonic fibroblasts and mesenchymal stem cells had increased levels of glycolytic enzymes when compared with wild type cells, and CAV1 silencing in these cells was sufficient to induce tumor growth when fibroblasts were cotransplanted with MDA‐MB‐231 xenografts.^127^ These studies led to formulate the “reverse Warburg effect” theory in CAFs.^128^ Proteomic analysis also provided first hints that CAV1‐silenced cells had enhanced oxidative stress by increasing levels of proteins related to this process, thus suggesting that CAV1 loss stabilized HIF1α through induction of reactive oxygen species production in a feed‐forward loop.^128^, ^129^, ^130^, ^131^ Accordingly, overexpression of antioxidant superoxide dismutase 2 in *Cav1*‐deficient fibroblasts counteracted their protumorigenic effect.^131^ In follow up studies, it was found that CAFs activated by oxidative stress increased secretion of lactate through upregulation of the lactate exporter MCT4.^132^, ^133^, ^134^ CAF‐secreted lactate was then taken up by surrounding cancer cells and converted into pyruvate, which fueled the tricarboxylic acid (TCA) cycle and oxidative phosphorylation to support biosynthetic processes and ATP production.^132^, ^134^

The above pioneering studies led to investigate reciprocal metabolic crosstalk between CAFs and cancer cells in other tumor models. In in vitro setups and syngeneic/xenograft models of subcutaneously/orthotopically cotransplanted pancreatic stellate cells (PSCs) and PDAC cells, cancer cells induced autophagy‐dependent secretion of alanine in PSCs. Alanine was taken up by cancer cells and converted into pyruvate to selectively support mitochondrial metabolism for the biosynthesis of non‐essential amino acids and lipids without affecting glycolysis. This metabolic crosstalk supported cancer cell proliferation under low nutrient culture conditions.^135^ High grade serous ovarian cancer cells (HeyA8 and SKOV3) secreted lactate, which was taken up by patient‐derived CAFs to fuel TCA cycle and support glutamine synthesis, in nutrient‐deprived conditions. CAF‐secreted glutamine was then used by cancer cells for nucleotide synthesis through the TCA cycle. Targeting glutamine synthetase in the stroma and glutaminase in the cancer cells blocked this metabolic symbiosis and reduced tumor growth, burden, and metastasis in a SKOV3 orthotopic tumor model.^136^ EVs secreted by patient‐derived prostate CAFs and pancreatic CAF‐19 line supported growth of pancreatic and prostate cancer cell lines via KRas‐independent mechanisms. CAF‐derived EVs taken up by cancer cells provided a wide range of metabolites and miRNAs, which decreased oxygen consumption rate, while enhanced glycolysis and reductive glutamine metabolism for fatty acid synthesis in the cancer cells.^137^

### 2.3 CAF Functions Revealed by Secretome Analysis

To identify CAF‐derived regulators of cancer cell proliferation, the CM of paired CAFs and normal fibroblasts isolated from colorectal adenocarcinoma and adjacent normal tissue were compared. Both cell types expressed α‐SMA and vimentin and their secretome had similar composition and anti‐proliferative functions. The proliferation of LoVo and HT‐29 cancer cells in vitro and the growth of LoVo xenografts in vivo decreased when cancer cells were cocultured or cotransplanted with CAFs or normal fibroblasts. The CM contained proteins related to ECM, adhesion, motility, inflammation, and proliferation. Among those, follistatin‐related protein 1 (FSTL1) and transgelin (TAGLN) controlled CAF antiproliferative function.^138^ In human gastric cancer with lymph node metastasis, low levels of the ECM adaptor protein like TGF‐β‐induced gene‐h3 were measured in the CAF secretome. TGF‐β‐induced gene‐h3 inhibits insulin‐like growth factor‐2‐stimulated cancer cell migration and proliferation, and growth of gastric cancer xenografts cotransplanted with fibroblasts.^117^ MS also unveiled the mechanisms underpinning the oncogenic effect of stromal *Pten*‐loss (see above). *Pten*‐loss in CAFs induced miR‐320 downregulation and this, in turn, increased the secretion of lysyl oxidase homolog 2 (LOXL2), bone morphogenetic protein 1, thrombospondin 1, matrix metalloproteinase‐2, MMP9, and elastin microfibril interface 2. The latter two promoted invasive behavior of DB7 cancer cells and ECs, respectively.^139^ MS‐secretomics and antibody array analyses of CM identified CAF‐derived regulators of colon cancer progression. Proteins released by patient‐derived CAFs were compared with those secreted by mesenchymal precursor cells (MSCs) derived from sternal bone marrow aspirates and TGF‐β‐treated MSCs. Proteins secreted by CAFs encompassed ECM components and modifiers, chemokines, growth factors, anti‐inflammatory/antioxidant, chaperones, metabolic, and cytoskeletal‐related proteins. The secretion of MMP3, Cathepsin D, HGF, tissue inhibitors of metalloproteinase‐1 (TIMP1), secreted protein acidic and rich in cysteine (SPARC), hyaluronan and proteoglycan link protein 1 (HAPLN1), GAL1, CXCL12, ED‐A, and TNC was confirmed in vitro using western blot and ELISA. A positive staining for the latter three was shown in the α‐SMA^+^ stroma of colon cancer patient samples.^140^ The comparison of CM from patient‐derived oral SCC CAFs with that of fibroblasts isolated from healthy oral mucosa showed that CAFs secrete more proteins related to collagen metabolism, ECM organization, and disassembly. RT‐qPCR and ELISA analyses confirmed increased levels of fibronectin type III domain‐containing 1 (FNDC1), serpin peptidase inhibitor type 1 (SERPINE1), and stanniocalcin 2 (STC2) in CAFs compared with normal fibroblasts across different CAF cell lines, and in normal fibroblasts upon TGF‐β‐stimulation.^141^ Our group has discovered that upon activation, CAFs secrete high levels of CLIC3, a protein that was previously known to be intracellular. CLIC3 was found to be a glutathione‐dependent oxidoreductase abundantly expressed in mammary and ovarian CAFs, as well as in the stroma of highly invasive breast and ovarian cancers. Extracellular CLIC3 enhanced vascularization of matrigel plugs subcutaneously implanted and invasion in MCF10DCIS.com xenografts through the activation of TGM2.^120^ In α‐SMA^+^ and FAP^+^ CT5.3 patient‐derived colon cancer CAFs, FAP expression decreased levels of the anti‐angiogenic pigment epithelium‐derived factor (PEDF) while increased ANG1 and VEGFC to promote angiogenesis and lymphangiogenesis. Accordingly, FAP‐silenced CAFs inhibited while FAP‐overexpressing CAFs promoted EC sprouting in vitro.^142^ Similarly, pharmacological inhibition of FAP activity in the syngeneic tumor model of CT26 colon cancer reduced tumor vascularization. Moreover, genetic deletion of FAP prior to tumor onset in KRas^G12D^‐driven model of lung adenocarcinoma and in CT26 tumors, as well as pharmacological inhibition of FAP, reduced cancer cell proliferation via activation of ECM/integrin‐mediated signaling.^143^ FAP also controlled the secretion of ADAMTS8 and MMP1, the cancer‐specific cleavage of collagens and other secreted proteins.^142^ To determine how CAFs contribute to the proteolysis of the tumor ECM, the CM of patient‐derived gastric CAFs was compared to that of adjacent tissue‐derived fibroblasts. Gastric CAFs upregulated the secretion of different MMPs. Using MS to identify fragments generated by proteolytic cleavage, MMP1 and MMP3 were found highly active in the secretome of CAFs. Enhanced levels and activity of MMPs were confirmed by western blot, enzymatic activity assays, and imaging techniques in CAFs cotransplanted with MKN45 gastric cancer cell xenografts. Functional assays with neutralizing antibodies and specific inhibitors for these proteases further demonstrated the pro‐migratory capacity of CAF‐derived MMPs on cancer cells.^144^ Dermal fibroblasts isolated from *Timp*‐deficient mice had a myofibroblast‐like phenotype and produced EVs with a different content compared with wild‐type fibroblasts. EVs from *Timp*‐deficient fibroblasts were enriched for ECM proteins, including LOXL2, TNC, and ADAM10. ADAM10‐rich EVs promoted motility of MDA‐MB‐231 cancer cells, and growth and metastasis of MDA‐MB‐231 xenografts. Similarly, ADAM10‐rich EVs from patient‐derived head and neck SCC CAFs promoted MDA‐MB‐231 migration.^145^ Overall, these studies highlight that the secretome is diverse in different CAFs; this may be a further indication of the heterogeneity and plasticity of these cells.

Also, the ECM derived from cells and tissues has been analyzed by MS (reviewed in Ref. 146); however, so far, only few studies have measured the ECM specifically secreted by fibroblasts^120^, ^147^ On the contrary, several works have analyzed the composition of the ECM in tumor tissues.^42^, ^148^, ^149^ Recently, such analysis has been performed also on ECM generated by an innovative approach of in situ decellularization of tissues (ISDot).^150^ Because in tissues the ECM can be produced by cancer and stromal cells, to address the cell‐specific contribution, human cancer cells can be transplanted into mice. This approach led to proof that, in melanoma xenografts, the tumor ECM was produced by both human cancer cells and murine stromal cells.^151^ From these studies, Naba and co‐workers have defined an accurate list of ECM proteins and proteins that can interact with the ECM or remodel it, which they have called “matrisome”.^151^

### 2.4 CAF Consensus Proteome and Secretome

**Figure 3:**
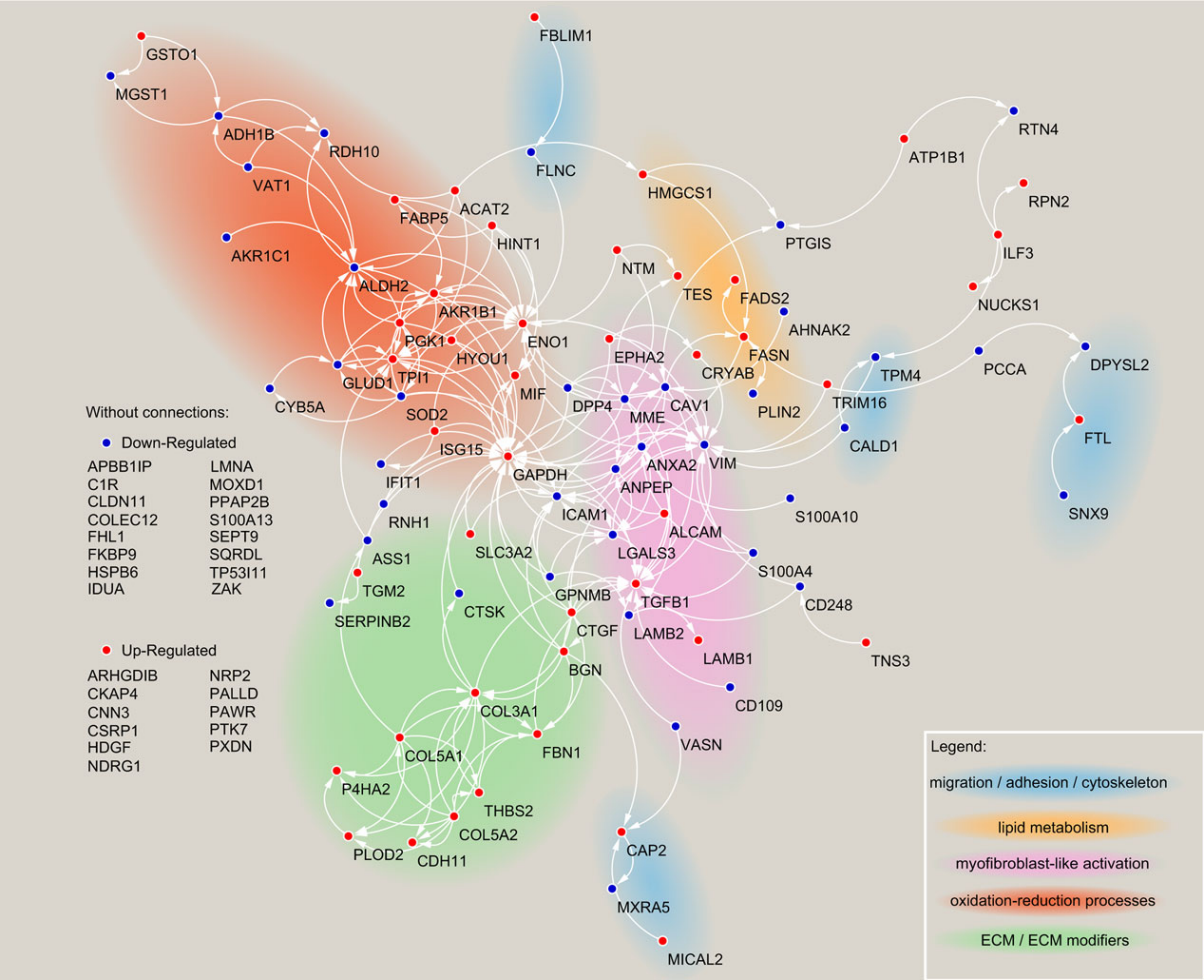
Consensus proteome. Proteins that were found consistently up‐ (red) and downregulated (blue) in the MS‐proteomic comparisons listed in Table 1 between matched CAFs and normal fibroblasts. Protein–protein interactions were define with STRING (version 10.5; all interaction sources were enabled and minimum interaction score of 0.4 was required) and visualized with Cytoscape.

**Figure 4:**
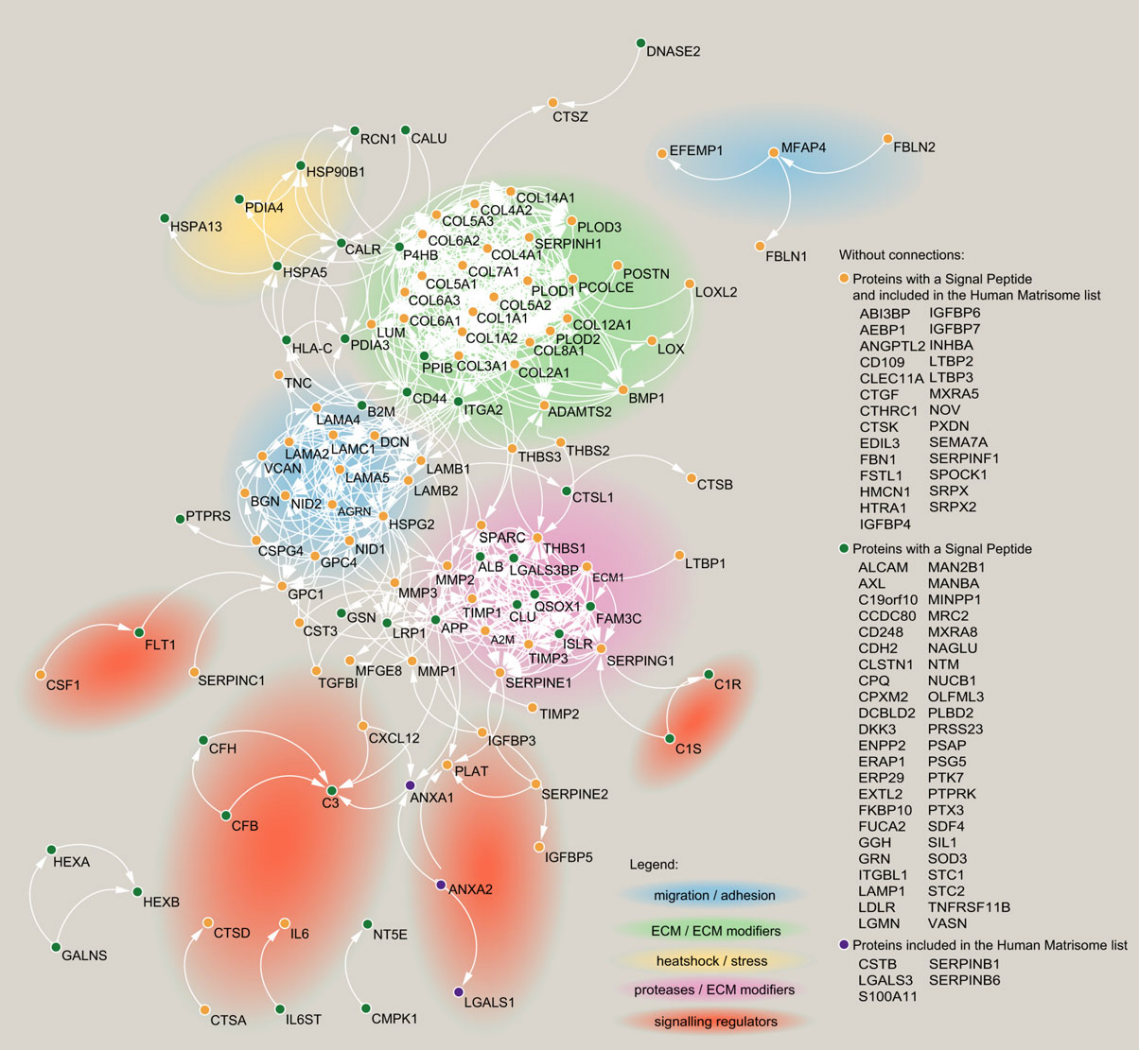
Consensus secretome. Secreted proteins that were consistently identified in the MS‐proteomic analysis of CAF‐derived conditioned media listed in Table 2. Protein–protein interactions were define with STRING (version 10.5; the active interaction sources were “experiments”, “databases”, and “co‐expression”, and minimum interaction score of 0.7 was required) and visualized with Cytoscape.

The lack of molecular markers unique for CAFs hindered the study of these cells. A key example is the challenge of isolating CAFs from tissues. The most used strategies are based on the isolation of cells expressing few known CAF markers, such as PDGFR or FAP, or by excluding cells expressing CD45, EpCAM, or Pecam1. However, CAFs are heterogeneous and using a single marker for their isolation may lead to the selection of a subpopulation only; negative selection allows isolation of heterogeneous CAFs, but could leave unwanted cells with the fibroblasts. Available proteomic data could be exploited to identify subsets of proteins consistently expressed by these cells that could help to establish panel of markers for their isolation and characterization. Moreover, since CAFs are highly secretory cells, a better characterization of their secretome could provide hints for prognostic and diagnostic markers. Identifying molecular markers specific for CAF subpopulations could also help to define their specialized secretory phenotype. Here we report an initial draft of the CAF consensus proteome (Figure 3) and secretome (Figure 4) based on the most in depth proteomic studies that we have found in the literature. For the consensus proteome we identified four studies which compared CAFs with their normal fibroblast counterpart (Table 1).^116^, ^117^, ^120^, ^121^ From each of those we considered the proteins differentially regulated between normal fibroblasts and CAFs as statistically assessed by the authors (only exception is Ref. 121: the original study reported only upregulated proteins, and here we report up and downregulated proteins based on a commonly used minimum fold change of two). Proteins regulated in at least half of the studies were used to build up a physical and functional interaction network with STRING^152^ (Figure 3). For the consensus secretome, we selected the proteins commonly identified in at least three out of the five studies (Table 2).^120^, ^138^, ^140^, ^141^, ^144^ To increase the confidence that those proteins are secreted, only the ones with a signal peptide (based on Uniprot^153^) and/or included in the human “matrisome”^154^ were included in the STRING network^152^ (Figure 4). This analysis highlighted several highly connected clusters, which were visualized with Cytoscape^155^ and annotated according to the function (literature‐based) of the proteins (Figures 3 and 4).

**Table 1:** Datasets used for the CAF proteome consensus signature in Figure 3

Tissue of origin (specie)	Quantification method	Number of proteins identified	Reference
Sporadic colon cancer (mouse)	Fourplex iTRAQ	1353	116
Gastric cancer (human)	Fourplex iTRAQ	768	117
Breast cancer (human)	SILAC	4113	120
Breast cancer (human)	Label‐free quantification	4094	121

**Table 2:** Datasets used for the CAF secretome consensus signature in Figure 4

CAF tissue of origin (specie)	Secretome enrichment strategy	Number of proteins identified	Reference
Colon cancer (human)	Ultrafiltration with Amicon Centriprep tubes YM‐3	367	140
Gastric cancer (human)	Ultrafiltration with Amicon Ultra 3 kDa	1460	144
Colon cancer (human)	Ultrafiltration with Amicon Ultra 3 kDa	227	138
Oral squamous cell carcinoma (human)	Ultrafiltration with Amicon Ultra 3 kDa	271	141
Mammary tumor (human)	Affinity based (Strataclean resin)	1527	120

## 3 Future Perspectives

CAFs have emerged as key players in the development of cancer pathology, but how these manipulators of the tumor microenvironment influence tumors is still largely unknown and rather controversial. Modern MS technology has the potential to provide valuable insights to answer this question, particularly to shed light on the reciprocal crosstalk between CAFs and other cell types in tumors and their heterogeneity.

Over the past decade, MS‐proteomics has broadened its applications for biology.^156^, ^157^ This has been achieved with the advances in MS technology which allow measuring proteomes and posttranslational modifications (PTMs) with high accuracy and to exceptional depth. Mass analyzers can now achieve unprecedented high resolution, such as the high field Orbitrap.^158^ Improved sample preparation prior to MS analysis also contributed to increase the coverage of the proteomes. Currently, high pH RP chromatography is a preferred method for peptide fractionation because it is orthogonal to the RP chromatography that is usually coupled on line to the mass spectrometer. This approach allows exceptional peptide separation resolution.^159^ Thanks to these improvements great depth can be achieved starting from sub‐milligram amounts of sample,^160^, ^161^ thus making MS a unique tool to assess complex biological systems that better mimic the in vivo situation, such as primary cells, heterotypic cultures, and cells cultured in 3D matrices. Also the robustness of protein quantification has improved. Software that use advanced algorithms^162^, ^163^ can accurately quantify peptides and PTMs using different quantification approaches, including stable isotope labeling with amino acids in cell culture (SILAC),^164^ tandem mass tag,^165^ and label free.

To unravel the crosstalk between CAFs and other cell types using heterotypic cultures, modern MS‐proteomics can be combined with cell‐type specific labeling with amino acid precursors (CTAP).^166^, ^167^ With this technique, cell type specific labeling is achieved through the cell specific expression of non‐mammalian enzymes that convert specific precursors into the essential amino acid l‐lysine. With CTAP cells are cultured for up to 7 days in the presence of heavy and light precursors. CTAP has recently unraveled the oncogenic KRas^G12D^‐induced reciprocal signaling that supports cancer cell proliferation and apoptosis in heterotypic co‐culture of PSCs and PDAC cells.^168^ Also SILAC can be used to assess reciprocal signaling in co‐cultured cells. In this case, cells labeled with heavy or light amino acids are co‐cultured in the absence of amino acids. For this reason, co‐cultures can last only for a short time, hours, to investigate dynamic signaling based on PTMs. SILAC also offers opportunities to investigate the transfer of proteins between CAFs and other cells. In co‐cultures, CAFs transfer proteins, lipids, and RNAs to cancer cells to influence their behavior.^114^, ^115^, ^169^ With Trans‐SILAC,^170^ it is possible to measure the repertoire of transferred proteins from SILAC‐labeled CAFs to other unlabeled cells in co‐cultures.

There are increasing evidences that CAF phenotypes are underpinned by epigenetic modifications,^171^ such as histone/DNA methylation and acetylation. Also CAF metabolism can control their activation.^172^ Epigenetics and metabolism can be tightly intertwined because Acetyl‐CoA and methionine provide building blocks for histone/DNA PTMs. MS‐proteomics has been recently used to trace the fate of stable isotope labeled metabolites into PTMs, thus offering a unique opportunity to explore the relationship between fibroblast metabolism, epigenetics, and phenotype.^173^

The mass cytometry by TOF (CyTOF) platform combines individual cell separation by flow cytometry with elemental metal isotope‐conjugated antibody (up to 40 antibody can be labeled in a single experiment). These labeled antibodies are used to label cells and can be detected by TOF mass analyzer.^174^, ^175^ CyTOF has been used to map cell subset–specific phenotypes of immune cells in clear cell renal carcinoma^176^ and early lung adenocarcinoma.^33^ A similar approach could help resolving the heterogeneity of CAF subpopulations in tumor tissues by using antibodies that detect known CAF markers, such as α‐SMA, FAP, FSP1/S100A4, and PDGFRs, or proteins found repeatedly induced in CAFs, such as those reported in Figure 3, and molecules that dictate their functions, such as IL‐6, CXCL12, and CLIC3. Once CAF subpopulations are identified, and strategies to isolate them from tumor tissues set up, in‐depth molecular and functional characterization can be performed. MS could also visualize the spatial distribution of CAF subpopulations within the stroma in intact tissue samples. MALDI coupled with TOF instruments^177^ has been the most utilized MS imaging technique to visualize proteins; tissues are embedded into a matrix and this allows efficient ionization of peptides and proteins (intact, top‐down, or digested, bottom‐up) for MS analysis. More recently nanospray desorption ESI^178^ coupled with high‐resolution mass analyzers has been used to identify peptides based on top‐down MS analysis.^179^ Both approaches can reach micrometer spatial resolution; however, nanospray desorption ESI has the advantage in that it requires minimal sample preparation before MS acquisition while MALDI achieves better ionization efficiency of protein and peptides.

**Figure 5:**
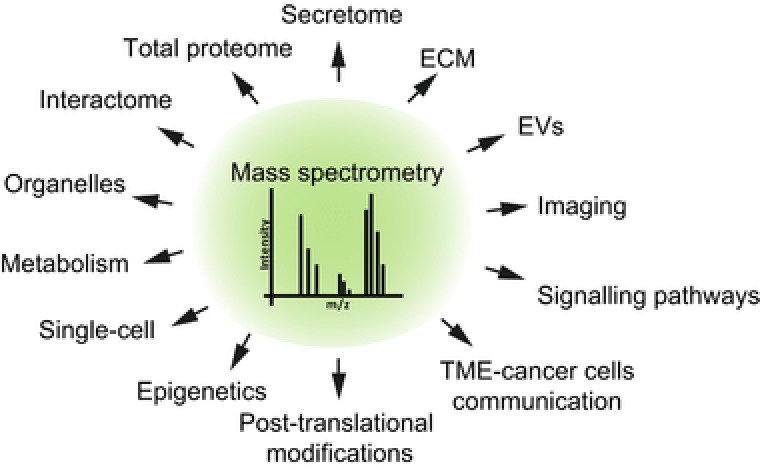
MS‐proteomics and CAFs. Scheme showing how MS‐proteomics can contribute to understand CAF biology. TME, tumor microenvironment.

In conclusion, the technological advances achieved over the past decade are opening exciting avenues to a deeper understanding of the biology of CAFs and contribute to provide insights to therapeutically target the stromal compartment to interfere with cancer progression and invasion (Figure 5).

**Abbreviations**
*α*‐SMA: alpha‐smooth muscle actin
ADAM: a disintegrin and metalloproteinase
ADAMTS: a disintegrin and metalloproteinase with thrombospondin motifs
ANGPT: angiopoietin
CAF: cancer associated fibroblast
CAV1: caveolin 1
CDH11: cadherin 11
CLIC: chloride intracellular channel
CM: conditioned medium
CTAP: cell‐type specific labeling with amino acid precursors
CXCL12: C‐X‐C motif chemokine 12
CyTOF: cytometry by TOF
EC: endothelial cell
ECM: extracellular matrix
EMT: epithelial to mesenchymal transition
EV: extracellular vesicle
FAP: fibroblast activation protein
FGF: fibroblast growth factor
FSP1/S100A4: fibroblasts‐specific protein 1
HIF1*α*: hypoxia‐inducible factor 1 alpha
IL: interleukin
LOX: lysyl oxidase
LTBP2: latent transforming growth factor beta binding protein 2
MMP: matrix metalloproteinase
NSCLC: non‐small cell lung cancer
P4HA: prolyl 4‐hydroxylase subunit alpha
PDAC: pancreatic adenocarcinoma
PDGF: platelet‐derived growth factor
PLOD2: procollagen‐lysine,2‐oxoglutarate 5‐dioxygenase 2
PTM: posttranslational modification
ROBO1: roundabout homolog 1
ROCK: Rho associated protein kinase
SCC: squamous cell carcinoma
SLC2A1: solute carrier family 2 facilitated glucose transportermember 1
TGF: transforming growth factor
TGM: transglutaminase
TIMP: tissue inhibitors of metalloproteinase
TME: tumor microenvironment
TNC: tenascin C
VEGF: vascular endothelial growth factor
YAP1: transcriptional coactivator YAP

## Conflict of Interest

The authors declare no conflict of interest.

## References

[1] D. Hanahan , R. A. Weinberg , Cell 2000, 100, 57. 10647931 10.1016/s0092-8674(00)81683-9

[2] D. Hanahan , R. A. Weinberg , Cell 2011, 144, 646. 21376230 10.1016/j.cell.2011.02.013

[3] T. Marsh , K. Pietras , S. S. McAllister , Biochim. Biophys. Acta 2013, 1832, 1070. 23123598 10.1016/j.bbadis.2012.10.013 PMC3775582

[4] G. Bergers , L. E. Benjamin , Nat. Rev. Cancer 2003, 3, 401. 12778130 10.1038/nrc1093

[5] F. Fan , A. Schimming , D. Jaeger , K. Podar , J. Oncol. 2012, 2012, 281261. 21876693 10.1155/2012/281261 PMC3163131

[6] O. Cleaver , D. A. Melton , Nat. Med. 2003, 9, 661. 12778164 10.1038/nm0603-661

[7] M. E. Ogle , C. E. Segar , S. Sridhar , E. A. Botchwey , Exp. Biol. Med. 2016, 241, 1084. 10.1177/1535370216650293 PMC4898192 27229903

[8] T. Asahara , H. Masuda , T. Takahashi , C. Kalka , C. Pastore , M. Silver , M. Kearne , M. Magner , J. M. Isner , Circ. Res. 1999, 85, 221. 10436164 10.1161/01.res.85.3.221

[9] D. H. Ausprunk , J. Folkman , Microvasc. Res. 1977, 14, 53. 895546 10.1016/0026-2862(77)90141-8

[10] F. Hillen , A. W. Griffioen , Cancer Metastasis Rev. 2007, 26, 489. 17717633 10.1007/s10555-007-9094-7 PMC2797856

[11] J. Holash , P. C. Maisonpierre , D. Compton , P. Boland , C. R. Alexander , D. Zagzag , G. D. Yancopoulos , S. J. Wiegand , Science 1999, 284, 1994. 10373119 10.1126/science.284.5422.1994

[12] S. Azzi , J. K. Hebda , J. Gavard , Front. Oncol. 2013, 3, 211. 23967403 10.3389/fonc.2013.00211 PMC3744053

[13] R. Kalluri , Nat. Rev. Cancer 2016, 16, 582. 27550820 10.1038/nrc.2016.73

[14] M. Papetti , I. M. Herman , Am. J. Physiol. Cell Physiol. 2002, 282, C947. 11940508 10.1152/ajpcell.00389.2001

[15] P. Baluk , H. Hashizume , D. M. McDonald , Curr. Opin. Genet. Dev. 2005, 15, 102. 15661540 10.1016/j.gde.2004.12.005

[16] E. di Tomaso , D. Capen , A. Haskell , J. Hart , J. J. Logie , R. K. Jain , D. M. McDonald , R. Jones , L. L. Munn , Cancer Res. 2005, 65, 5740. 15994949 10.1158/0008-5472.CAN-04-4552

[17] A. C. Dudley , Cold Spring Harb. Perspect. Med. 2012, 2, a006536. 22393533 10.1101/cshperspect.a006536 PMC3282494

[18] M. A. Konerding , W. Malkusch , B. Klapthor , C. van Ackern , E. Fait , S. A. Hill , C. Parkins , D. J. Chaplin , M. Presta , J. Denekamp , Br. J. Cancer 1999, 80, 724. 10360650 10.1038/sj.bjc.6690416 PMC2362271

[19] B. A. Warren , P. Shubik , R. Wilson , H. Garcia , R. Feldman , Cancer Lett. 1978, 4, 109. 647650 10.1016/s0304-3835(78)93797-7

[20] T. P. Padera , B. R. Stoll , J. B. Tooredman , D. Capen , E. di Tomaso , R. K. Jain , Nature 2004, 427, 695. 14973470 10.1038/427695a

[21] N. Reymond , B. B. d'Agua , A. J. Ridley , Nat. Rev. Cancer 2013, 13, 858. 24263189 10.1038/nrc3628

[22] K. Castermans , A. W. Griffioen , Biochim. Biophys. Acta 2007, 1776, 160. 17888580 10.1016/j.bbcan.2007.07.005

[23] A. McIntyre , A. L. Harris , EMBO Mol. Med. 2015, 7, 368. 25700172 10.15252/emmm.201404271 PMC4403040

[24] T. Hoshida , N. Isaka , J. Hagendoorn , E. di Tomaso , Y. L. Chen , B. Pytowski , D. Fukumura , T. P. Padera , R. K. Jain , Cancer Res. 2006, 66, 8065. 16912183 10.1158/0008-5472.CAN-06-1392

[25] T. Karpanen , M. Egeblad , M. J. Karkkainen , H. Kubo , S. Yla‐Herttuala , M. Jaattela , K. Alitalo , Cancer Res. 2001, 61, 1786. 11280723

[26] A. J. Leu , D. A. Berk , A. Lymboussaki , K. Alitalo , R. K. Jain , Cancer Res. 2000, 60, 4324. 10969769

[27] S. J. Mandriota , L. Jussila , M. Jeltsch , A. Compagni , D. Baetens , R. Prevo , S. Banerji , J. Huarte , R. Montesano , D. G. Jackson , L. Orci , K. Alitalo , G. Christofori , M. S. Pepper , EMBO J. 2001, 20, 672. 11179212 10.1093/emboj/20.4.672 PMC145430

[28] K. Alitalo , Nat. Med. 2011, 17, 1371. 22064427 10.1038/nm.2545

[29] W. Debinski , B. Slagle‐Webb , M. G. Achen , S. A. Stacker , E. Tulchinsky , G. Y. Gillespie , D. M. Gibo , Mol. Med. 2001, 7, 598. 11778649 PMC1950071

[30] S. F. Schoppmann , P. Birner , J. Stockl , R. Kalt , R. Ullrich , C. Caucig , E. Kriehuber , K. Nagy , K. Alitalo , D. Kerjaschki , Am. J. Pathol. 2002, 161, 947. 12213723 10.1016/S0002-9440(10)64255-1 PMC1867252

[31] G. Dranoff , Nat. Rev. Cancer 2004, 4, 11–22. 14708024 10.1038/nrc1252

[32] Y. Lavin , D. Winter , R. Blecher‐Gonen , E. David , H. Keren‐Shaul , M. Merad , S. Jung , I. Amit , Cell 2014, 159, 1312. 25480296 10.1016/j.cell.2014.11.018 PMC4437213

[33] Y. Lavin , S. Kobayashi , A. Leader , E. D. Amir , N. Elefant , C. Bigenwald , R. Remark , R. Sweeney , C. D. Becker , J. H. Levine , K. Meinhof , A. Chow , S. Kim‐Shulze , A. Wolf , C. Medaglia , H. Li , J. A. Rytlewski , R. O. Emerson , A. Solovyov , B. D. Greenbaum , C. Sanders , M. Vignali , M. B. Beasley , R. Flores , S. Gnjatic , D. Pe'er , A. Rahman , I. Amit , M. Merad , Cell 2017, 169, 750 e717. 28475900 10.1016/j.cell.2017.04.014 PMC5737939

[34] D. E. Speiser , P. C. Ho , G. Verdeil , Nat. Rev. Immunol. 2016, 16, 599. 27526640 10.1038/nri.2016.80

[35] P. Sharma , J. P. Allison , Cell 2015, 161, 205. 25860605 10.1016/j.cell.2015.03.030 PMC5905674

[36] D. Nummer , E. Suri‐Payer , H. Schmitz‐Winnenthal , A. Bonertz , L. Galindo , D. Antolovich , M. Koch , M. Buchler , J. Weitz , V. Schirrmacher , P. Beckhove , J. Natl. Cancer Inst. 2007, 99, 1188. 17652277 10.1093/jnci/djm064

[37] S. Shetty , C. J. Weston , Y. H. Oo , N. Westerlund , Z. Stamataki , J. Youster , S. G. Hubscher , M. Salmi , S. Jalkanen , P. F. Lalor , D. H. Adams , J. Immunol. 2011, 186, 4147. 21368224 10.4049/jimmunol.1002961 PMC6016742

[38] M. Egeblad , M. G. Rasch , V. M. Weaver , Curr. Opin. Cell Biol. 2010, 22, 697. 20822891 10.1016/j.ceb.2010.08.015 PMC2948601

[39] S. Ozbek , P. G. Balasubramanian , R. Chiquet‐Ehrismann , R. P. Tucker , J. C. Adams , Mol. Biol. Cell 2010, 21, 4300. 21160071 10.1091/mbc.E10-03-0251 PMC3002383

[40] T. Rozario , D. W. DeSimone , Dev. Biol. 2010, 341, 126. 19854168 10.1016/j.ydbio.2009.10.026 PMC2854274

[41] S. A. Wickstrom , K. Radovanac , R. Fassler , Cold Spring Harb. Perspect. Biol. 2011, 3. 10.1101/cshperspect.a005116 PMC3039529 21421914

[42] A. Naba , K. R. Clauser , J. M. Lamar , S. A. Carr , R. O. Hynes , Elife 2014, 3, e01308. 24618895 10.7554/eLife.01308 PMC3944437

[43] L. Perryman , J. T. Erler , Future Oncol. 2014, 10, 1709. 25145437 10.2217/fon.14.39

[44] Z. Wang , M. Griffin , Amino Acids 2012, 42, 939. 21818567 10.1007/s00726-011-1008-x

[45] D. R. Edwards , M. M. Handsley , C. J. Pennington , Mol. Aspects Med. 2008, 29, 258. 18762209 10.1016/j.mam.2008.08.001 PMC7112278

[46] G. Murphy , H. Nagase , Mol. Aspects Med. 2008, 29, 290. 18619669 10.1016/j.mam.2008.05.002 PMC2810947

[47] S. Porter , I. M. Clark , L. Kevorkian , D. R. Edwards , Biochem. J. 2005, 386, 15. 15554875 10.1042/BJ20040424 PMC1134762

[48] K. Brew , H. Nagase , Biochim. Biophys. Acta 2010, 1803, 55. 20080133 10.1016/j.bbamcr.2010.01.003 PMC2853873

[49] P. Lu , V. M. Weaver , Z. Werb , J. Cell Biol. 2012, 196, 395. 22351925 10.1083/jcb.201102147 PMC3283993

[50] M. W. Pickup , J. K. Mouw , V. M. Weaver , EMBO Rep. 2014, 15, 1243. 25381661 10.15252/embr.201439246 PMC4264927

[51] I. Acerbi , L. Cassereau , I. Dean , Q. Shi , A. Au , C. Park , Y. Y. Chen , J. Liphardt , E. S. Hwang , V. M. Weaver , Integr. Biol. 2015, 7, 1120. 10.1039/c5ib00040h PMC4593730 25959051

[52] F. Calvo , N. Ege , A. Grande‐Garcia , S. Hooper , R. P. Jenkins , S. I. Chaudhry , K. Harrington , P. Williamson , E. Moeendarbary , G. Charras , E. Sahai , Nat. Cell Biol. 2013, 15, 637. 23708000 10.1038/ncb2756 PMC3836234

[53] S. E. Reid , E. J. Kay , L. J. Neilson , A. T. Henze , J. Serneels , E. J. McGhee , S. Dhayade , C. Nixon , J. B. Mackey , A. Santi , K. Swaminathan , D. Athineos , V. Papalazarou , F. Patella , A. Roman‐Fernandez , Y. ElMaghloob , J. R. Hernandez‐Fernaud , R. H. Adams , S. Ismail , D. M. Bryant , M. Salmeron‐Sanchez , L. M. Machesky , L. M. Carlin , K. Blyth , M. Mazzone , S. Zanivan , EMBO J., 2017, 36, 2373. 28694244 10.15252/embj.201694912 PMC5556271

[54] M. H. Zaman , L. M. Trapani , A. L. Sieminski , D. Mackellar , H. Gong , R. D. Kamm , A. Wells , D. A. Lauffenburger , P. Matsudaira , PNAS 2006, 103, 10889. 16832052 10.1073/pnas.0604460103 PMC1544144

[55] M. Jacob , L. Chang , E. Pure , Curr. Mol. Med. 2012, 12, 1220. 22834826 10.2174/156652412803833607

[56] R. Kalluri , Nat. Rev. Cancer 2003, 3, 422. 12778132 10.1038/nrc1094

[57] G. Gabbiani , G. B. Ryan , G. Majne , Experientia 1971, 27, 549. 5132594 10.1007/BF02147594

[58] G. Serini , M. L. Bochaton‐Piallat , P. Ropraz , A. Geinoz , L. Borsi , L. Zardi , G. Gabbiani , J. Cell Biol. 1998, 142, 873. 9700173 10.1083/jcb.142.3.873 PMC2148176

[59] G. Gabbiani , J. Pathol. 2003, 200, 500. 12845617 10.1002/path.1427

[60] G. Tremblay , Exp. Mol. Pathol. 1979, 31, 248. 222605 10.1016/0014-4800(79)90026-1

[61] L. Ronnov‐Jessen , O. W. Petersen , Lab. Invest. 1993, 68, 696. 8515656

[62] N. C. Direkze , M. R. Alison , Hematol. Oncol. 2006, 24, 189. 16795113 10.1002/hon.788

[63] P. Cirri , P. Chiarugi , Am. J. Cancer Res. 2011, 1, 482. 21984967 PMC3186047

[64] D. C. Radisky , P. A. Kenny , M. J. Bissell , J. Cell. Biochem. 2007, 101, 830. 17211838 10.1002/jcb.21186 PMC2838476

[65] E. M. Zeisberg , S. Potenta , L. Xie , M. Zeisberg , R. Kalluri , Cancer Res. 2007, 67, 10123. 17974953 10.1158/0008-5472.CAN-07-3127

[66] E. L. Spaeth , J. L. Dembinski , A. K. Sasser , K. Watson , A. Klopp , B. Hall , M. Andreeff , F. Marini , PLoS One 2009, 4, e4992. 19352430 10.1371/journal.pone.0004992 PMC2661372

[67] L. Bochet , C. Lehuede , S. Dauvillier , Y. Y. Wang , B. Dirat , V. Laurent , C. Dray , R. Guiet , I. Maridonneau‐Parini , S. Le Gonidec , B. Couderc , G. Escourrou , P. Valet , C. Muller , Cancer Res. 2013, 73, 5657. 23903958 10.1158/0008-5472.CAN-13-0530

[68] P. J. Barth , S. Ebrahimsade , A. Ramaswamy , R. Moll , Virchows Arch. 2002, 440, 298. 11889601 10.1007/s004280100530

[69] R. Kalluri , M. Zeisberg , Nat. Rev. Cancer 2006, 6, 392. 16572188 10.1038/nrc1877

[70] O. W. Petersen , H. L. Nielsen , T. Gudjonsson , R. Villadsen , F. Rank , E. Niebuhr , M. J. Bissell , L. Ronnov‐Jessen , Am. J. Pathol. 2003, 162, 391. 12547698 10.1016/S0002-9440(10)63834-5 PMC1851146

[71] O. De Wever , P. Demetter , M. Mareel , M. Bracke , Int. J. Cancer 2008, 123, 2229. 18777559 10.1002/ijc.23925

[72] D. A. Bronzert , P. Pantazis , H. N. Antoniades , A. Kasid , N. Davidson , R. B. Dickson , M. E. Lippman , PNAS 1987, 84, 5763. 3039506 10.1073/pnas.84.16.5763 PMC298943

[73] E. Giannoni , F. Bianchini , L. Masieri , S. Serni , E. Torre , L. Calorini , P. Chiarugi , Cancer Res. 2010, 70, 6945. 20699369 10.1158/0008-5472.CAN-10-0785

[74] M. Lohr , C. Schmidt , J. Ringel , M. Kluth , P. Muller , H. Nizze , R. Jesnowski , Cancer Res. 2001, 61, 550. 11212248

[75] Z. M. Shao , M. Nguyen , S. H. Barsky , Oncogene 2000, 19, 4337. 10980609 10.1038/sj.onc.1203785

[76] F. Strutz , M. Zeisberg , B. Hemmerlein , B. Sattler , K. Hummel , V. Becker , G. A. Muller , Kidney Int. 2000, 57, 1521. 10760088 10.1046/j.1523-1755.2000.00997.x

[77] U. E. Martinez‐Outschoorn , S. Pavlides , D. Whitaker‐Menezes , K. M. Daumer , J. N. Milliman , B. Chiavarina , G. Migneco , A. K. Witkiewicz , M. P. Martinez‐Cantarin , N. Flomenberg , A. Howell , R. G. Pestell , M. P. Lisanti , F. Sotgia , Cell Cycle 2010, 9, 2423. 20562526 10.4161/cc.9.12.12048

[78] A. Toullec , D. Gerald , G. Despouy , B. Bourachot , M. Cardon , S. Lefort , M. Richardson , G. Rigaill , M. C. Parrini , C. Lucchesi , D. Bellanger , M. H. Stern , T. Dubois , X. Sastre‐Garau , O. Delattre , A. Vincent‐Salomon , F. Mechta‐Grigoriou , EMBO Mol. Med. 2010, 2, 211. 20535745 10.1002/emmm.201000073 PMC3377319

[79] P. Gascard , T. D. Tlsty , Genes Dev. 2016, 30, 1002. 27151975 10.1101/gad.279737.116 PMC4863733

[80] H. Sugimoto , T. M. Mundel , M. W. Kieran , R. Kalluri , Cancer Biol. Ther. 2006, 5, 1640. 17106243 10.4161/cbt.5.12.3354

[81] D. Ohlund , A. Handly‐Santana , G. Biffi , E. Elyada , A. S. Almeida , M. Ponz‐Sarvise , V. Corbo , T. E. Oni , S. A. Hearn , E. J. Lee , I. I. Chio , C. I. Hwang , H. Tiriac , L. A. Baker , D. D. Engle , C. Feig , A. Kultti , M. Egeblad , D. T. Fearon , J. M. Crawford , H. Clevers , Y. Park , D. A. Tuveson , J. Exp. Med. 2017, 214, 579. 28232471 10.1084/jem.20162024 PMC5339682

[82] C. J. Hanley , F. Noble , M. Ward , M. Bullock , C. Drifka , M. Mellone , A. Manousopoulou , H. E. Johnston , A. Hayden , S. Thirdborough , Y. Liu , D. M. Smith , T. Mellows , W. J. Kao , S. D. Garbis , A. Mirnezami , T. J. Underwood , K. W. Eliceiri , G. J. Thomas , Oncotarget 2016, 7, 6159 26716418 10.18632/oncotarget.6740 PMC4868747

[83] E. Hirata , M. R. Girotti , A. Viros , S. Hooper , B. Spencer‐Dene , M. Matsuda , J. Larkin , R. Marais , E. Sahai , Cancer Cell 2015, 27, 574 25873177 10.1016/j.ccell.2015.03.008 PMC4402404

[84] R. Straussman , T. Morikawa , K. Shee , M. Barzily‐Rokni , Z. R. Qian , J. Du , A. Davis , M. M. Mongare , J. Gould , D. T. Frederick , Z. A. Cooper , P. B. Chapman , D. B. Solit , A. Ribas , R. S. Lo , K. T. Flaherty , S. Ogino , J. A. Wargo , T. R. Golub , Nature 2012, 487, 500 22763439 10.1038/nature11183 PMC3711467

[85] C. Gaggioli , S. Hooper , C. Hidalgo‐Carcedo , R. Grosse , J. F. Marshall , K. Harrington , E. Sahai , Nat. Cell Biol. 2007, 9, 1392 18037882 10.1038/ncb1658

[86] V. Sanz‐Moreno , C. Gaggioli , M. Yeo , J. Albrengues , F. Wallberg , A. Viros , S. Hooper , R. Mitter , C. C. Feral , M. Cook , J. Larkin , R. Marais , G. Meneguzzi , E. Sahai , C. J. Marshall , Cancer Cell 2011, 20, 229 21840487 10.1016/j.ccr.2011.06.018

[87] C. Vennin , N. Rath , M. Pajic , M. F. Olson , P. Timpson , Small GTPases, 2017, 3, 1, https://doi.org/10.1080/21541248.2017. 10.1080/21541248.2017.1345712 PMC6959285 28972449

[88] C. J. Whatcott , S. Ng , M. T. Barrett , G. Hostetter , D. D. Von Hoff , H. Han , PLoS One 2017, 12, e0183871 28841710 10.1371/journal.pone.0183871 PMC5571985

[89] X. Tang , Y. Hou , G. Yang , X. Wang , S. Tang , Y. E. Du , L. Yang , T. Yu , H. Zhang , M. Zhou , S. Wen , L. Xu , M. Liu , Cell Death Differ. 2016, 23, 132 26068592 10.1038/cdd.2015.78 PMC4815985

[90] D. M. Gilkes , G. L. Semenza , D. Wirtz , Nat. Rev. Cancer 2014, 14, 430 24827502 10.1038/nrc3726 PMC4283800

[91] D. M. Gilkes , S. Bajpai , P. Chaturvedi , D. Wirtz , G. L. Semenza , J. Biol. Chem. 2013, 288, 10819 23423382 10.1074/jbc.M112.442939 PMC3624462

[92] B. Chiavarina , D. Whitaker‐Menezes , G. Migneco , U. E. Martinez‐Outschoorn , S. Pavlides , A. Howell , H. B. Tanowitz , M. C. Casimiro , C. Wang , R. G. Pestell , P. Grieshaber , J. Caro , F. Sotgia , M. P. Lisanti , Cell Cycle 2010, 9, 3534 20864819 10.4161/cc.9.17.12908 PMC3047618

[93] C. D. Madsen , J. T. Pedersen , F. A. Venning , L. B. Singh , E. Moeendarbary , G. Charras , T. R. Cox , E. Sahai , J. T. Erler , EMBO Rep. 2015, 16, 1394 26323721 10.15252/embr.201540107 PMC4662858

[94] J. W. Kim , C. Evans , A. Weidemann , N. Takeda , Y. S. Lee , C. Stockmann , C. Branco‐Price , F. Brandberg , G. Leone , M. C. Ostrowski , R. S. Johnson , Cancer Res. 2012, 72, 3187 22556263 10.1158/0008-5472.CAN-12-0534 PMC4089958

[95] A. Kuchnio , S. Moens , U. Bruning , K. Kuchnio , B. Cruys , B. Thienpont , M. Broux , A. A. Ungureanu , R. Leite de Oliveira , F. Bruyere , H. Cuervo , A. Manderveld , A. Carton , J. R. Hernandez‐Fernaud , S. Zanivan , C. Bartic , J. M. Foidart , A. Noel , S. Vinckier , D. Lambrechts , M. Dewerchin , M. Mazzone , P. Carmeliet , Cell Rep 2015, 12, 992 26235614 10.1016/j.celrep.2015.07.010

[96] P. S. Soon , E. Kim , C. K. Pon , A. J. Gill , K. Moore , A. J. Spillane , D. E. Benn , R. C. Baxter , Endocr. Relat. Cancer 2013, 20, 1 23111755 10.1530/ERC-12-0227

[97] Y. Yu , C. H. Xiao , L. D. Tan , Q. S. Wang , X. Q. Li , Y. M. Feng , Br. J. Cancer 2014, 110, 724 24335925 10.1038/bjc.2013.768 PMC3915130

[98] Y. Shintani , A. Fujiwara , T. Kimura , T. Kawamura , S. Funaki , M. Minami , M. Okumura , J. Thor, Oncol. 2016, 11, 1482 10.1016/j.jtho.2016.05.025 27287412

[99] Y. Chong , D. Tang , Q. Xiong , X. Jiang , C. Xu , Y. Huang , J. Wang , H. Zhou , Y. Shi , X. Wu , D. Wang , J. Exp. Clin. Cancer Res. 2016, 35, 175 27836001 10.1186/s13046-016-0449-1 PMC5106768

[100] A. Labernadie , T. Kato , A. Brugues , X. Serra‐Picamal , S. Derzsi , E. Arwert , A. Weston , V. Gonzalez‐Tarrago , A. Elosegui‐Artola , L. Albertazzi , J. Alcaraz , P. Roca‐Cusachs , E. Sahai , X. Trepat , Nat. Cell Biol. 2017, 19, 224 28218910 10.1038/ncb3478 PMC5831988

[101] D. G. Duda , A. M. Duyverman , M. Kohno , M. Snuderl , E. J. Steller , D. Fukumura , R. K. Jain , PNAS 2010, 107, 21677 21098274 10.1073/pnas.1016234107 PMC3003109

[102] B. C. Ozdemir , T. Pentcheva‐Hoang , J. L. Carstens , X. Zheng , C. C. Wu , T. R. Simpson , H. Laklai , H. Sugimoto , C. Kahlert , S. V. Novitskiy , A. De Jesus‐Acosta , P. Sharma , P. Heidari , U. Mahmood , L. Chin , H. L. Moses , V. M. Weaver , A. Maitra , J. P. Allison , V. S. LeBleu , R. Kalluri , Cancer Cell 2014, 25, 719 24856586 10.1016/j.ccr.2014.04.005 PMC4180632

[103] A. D. Rhim , P. E. Oberstein , D. H. Thomas , E. T. Mirek , C. F. Palermo , S. A. Sastra , E. N. Dekleva , T. Saunders , C. P. Becerra , I. W. Tattersall , C. B. Westphalen , J. Kitajewski , M. G. Fernandez‐Barrena , M. E. Fernandez‐Zapico , C. Iacobuzio‐Donahue , K. P. Olive , B. Z. Stanger , Cancer Cell 2014, 25, 735 24856585 10.1016/j.ccr.2014.04.021 PMC4096698

[104] P. H. Chang , W. W. Hwang‐Verslues , Y. C. Chang , C. C. Chen , M. Hsiao , Y. M. Jeng , K. J. Chang , E. Y. Lee , J. Y. Shew , W. H. Lee , Cancer Res. 2012, 72, 4652 22826604 10.1158/0008-5472.CAN-12-0877 PMC3445732

[105] P. Maris , A. Blomme , A. P. Palacios , B. Costanza , A. Bellahcene , E. Bianchi , S. Gofflot , P. Drion , G. E. Trombino , E. Di Valentin , P. G. Cusumano , S. Maweja , G. Jerusalem , P. Delvenne , E. Lifrange , V. Castronovo , A. Turtoi , PLoS Med 2015, 12, e1001871 26327350 10.1371/journal.pmed.1001871 PMC4556693

[106] D. Fukumura , R. Xavier , T. Sugiura , Y. Chen , E. C. Park , N. Lu , M. Selig , G. Nielsen , T. Taksir , R. K. Jain , B. Seed , Cell 1998, 94, 715 9753319 10.1016/s0092-8674(00)81731-6

[107] K. Pietras , J. Pahler , G. Bergers , D. Hanahan , PLoS Med 2008, 5, e19 18232728 10.1371/journal.pmed.0050019 PMC2214790

[108] A. Orimo , P. B. Gupta , D. C. Sgroi , F. Arenzana‐Seisdedos , T. Delaunay , R. Naeem , V. J. Carey , A. L. Richardson , R. A. Weinberg , Cell 2005, 121, 335 15882617 10.1016/j.cell.2005.02.034

[109] A. J. Trimboli , C. Z. Cantemir‐Stone , F. Li , J. A. Wallace , A. Merchant , N. Creasap , J. C. Thompson , E. Caserta , H. Wang , J. L. Chong , S. Naidu , G. Wei , S. M. Sharma , J. A. Stephens , S. A. Fernandez , M. N. Gurcan , M. B. Weinstein , S. H. Barsky , L. Yee , T. J. Rosol , P. C. Stromberg , M. L. Robinson , F. Pepin , M. Hallett , M. Park , M. C. Ostrowski , G. Leone , Nature 2009, 461, 1084 19847259 10.1038/nature08486 PMC2767301

[110] N. Erez , M. Truitt , P. Olson , S. T. Arron , D. Hanahan , Cancer Cell 2010, 17, 135 20138012 10.1016/j.ccr.2009.12.041

[111] P. Chomarat , J. Banchereau , J. Davoust , A. K. Palucka , Nat. Immunol. 2000, 1, 510 11101873 10.1038/82763

[112] D. Liao , Y. Luo , D. Markowitz , R. Xiang , R. A. Reisfeld , PLoS One 2009, 4, e7965 19956757 10.1371/journal.pone.0007965 PMC2775953

[113] C. Feig , J. O. Jones , M. Kraman , R. J. Wells , A. Deonarine , D. S. Chan , C. M. Connell , E. W. Roberts , Q. Zhao , O. L. Caballero , S. A. Teichmann , T. Janowitz , D. I. Jodrell , D. A. Tuveson , D. T. Fearon , PNAS 2013, 110, 20212 24277834 10.1073/pnas.1320318110 PMC3864274

[114] M. C. Boelens , T. J. Wu , B. Y. Nabet , B. Xu , Y. Qiu , T. Yoon , D. J. Azzam , C. Twyman‐Saint\sVictor , B. Z. Wiemann , H. Ishwaran , P. J. Ter Brugge , J. Jonkers , J. Slingerland , A. J. Minn , Cell 2014, 159, 499 25417103 10.1016/j.cell.2014.09.051 PMC4283810

[115] B. Y. Nabet , Y. Qiu , J. E. Shabason , T. J. Wu , T. Yoon , B. C. Kim , J. L. Benci , A. M. DeMichele , J. Tchou , J. Marcotrigiano , A. J. Minn , Cell 2017, 170, 352–366 e313 10.1016/j.cell.2017.06.031 PMC6611169 28709002

[116] S. Torres , R. A. Bartolome , M. Mendes , R. Barderas , M. J. Fernandez‐Acenero , A. Pelaez‐Garcia , C. Pena , M. Lopez‐Lucendo , R. Villar‐Vazquez , A. G. de Herreros , F. Bonilla , J. I. Casal , Clin. Cancer Res. 2013, 19, 6006 24025712 10.1158/1078-0432.CCR-13-1130

[117] C. Holmberg , M. Quante , I. Steele , J. D. Kumar , S. Balabanova , C. Duval , M. Czepan , Z. Rakonczay, Jr. , L. Tiszlavicz , I. Nemeth , G. Lazar , Z. Simonka , R. Jenkins , P. Hegyi , T. C. Wang , G. J. Dockray , A. Varro , Carcinogenesis 2012, 33, 1553 22610072 10.1093/carcin/bgs180 PMC3499060

[118] N. M. Hawsawi , H. Ghebeh , S. F. Hendrayani , A. Tulbah , M. Al‐Eid , T. Al‐Tweigeri , D. Ajarim , A. Alaiya , S. Dermime , A. Aboussekhra , Cancer Res. 2008, 68, 2717 18413739 10.1158/0008-5472.CAN-08-0192

[119] Y. Kojima , A. Acar , E. N. Eaton , K. T. Mellody , C. Scheel , I. Ben‐Porath , T. T. Onder , Z. C. Wang , A. L. Richardson , R. A. Weinberg , A. Orimo , PNAS 2010, 107, 20009 21041659 10.1073/pnas.1013805107 PMC2993333

[120] J. R. Hernandez‐Fernaud , E. Ruengeler , A. Casazza , L. J. Neilson , E. Pulleine , A. Santi , S. Ismail , S. Lilla , S. Dhayade , I. R. MacPherson , I. McNeish , D. Ennis , H. Ali , F. G. Kugeratski , H. Al Khamici , M. van den Biggelaar , P. V. van den Berghe , C. Cloix , L. McDonald , D. Millan , A. Hoyle , A. Kuchnio , P. Carmeliet , S. M. Valenzuela , K. Blyth , H. Yin , M. Mazzone , J. C. Norman , S. Zanivan , Nat. Commun. 2017, 8, 14206 28198360 10.1038/ncomms14206 PMC5316871

[121] M. Groessl , A. Slany , A. Bileck , K. Gloessmann , D. Kreutz , W. Jaeger , G. Pfeiler , C. Gerner , J. Proteome Res. 2014, 13, 4773 25238572 10.1021/pr500727h

[122] A. Slany , V. Haudek‐Prinz , H. Zwickl , S. Stattner , B. Grasl‐Kraupp , C. Gerner , Electrophoresis 2013, 34, 3315 24115093 10.1002/elps.201300326

[123] C. Guido , D. Whitaker‐Menezes , Z. Lin , R. G. Pestell , A. Howell , T. A. Zimmers , M. C. Casimiro , S. Aquila , S. Ando , U. E. Martinez‐Outschoorn , F. Sotgia , M. P. Lisanti , Oncotarget 2012, 3, 798 22878233 10.18632/oncotarget.574 PMC3478457

[124] I. Mercier , M. C. Casimiro , C. Wang , A. L. Rosenberg , J. Quong , A. Minkeu , K. G. Allen , C. Danilo , F. Sotgia , G. Bonuccelli , J. F. Jasmin , H. Xu , E. Bosco , B. Aronow , A. Witkiewicz , R. G. Pestell , E. S. Knudsen , M. P. Lisanti , Cancer Biol. Ther. 2008, 7, 1212 18458534 10.4161/cbt.7.8.6220 PMC6688494

[125] J. Pouyssegur , F. Mechta‐Grigoriou , Biol. Chem. 2006, 387, 1337 17081104 10.1515/BC.2006.167

[126] U. E. Martinez‐Outschoorn , C. Trimmer , Z. Lin , D. Whitaker‐Menezes , B. Chiavarina , J. Zhou , C. Wang , S. Pavlides , M. P. Martinez‐Cantarin , F. Capozza , A. K. Witkiewicz , N. Flomenberg , A. Howell , R. G. Pestell , J. Caro , M. P. Lisanti , F. Sotgia , Cell Cycle 2010, 9, 3515 20855962 10.4161/cc.9.17.12928 PMC3047617

[127] G. Bonuccelli , D. Whitaker‐Menezes , R. Castello‐Cros , S. Pavlides , R. G. Pestell , A. Fatatis , A. K. Witkiewicz , M. G. Vander Heiden , G. Migneco , B. Chiavarina , P. G. Frank , F. Capozza , N. Flomenberg , U. E. Martinez‐Outschoorn , F. Sotgia , M. P. Lisanti , Cell Cycle 2010, 9, 1960 20495363 10.4161/cc.9.10.11601

[128] S. Pavlides , D. Whitaker‐Menezes , R. Castello‐Cros , N. Flomenberg , A. K. Witkiewicz , P. G. Frank , M. C. Casimiro , C. Wang , P. Fortina , S. Addya , R. G. Pestell , U. E. Martinez‐Outschoorn , F. Sotgia , M. P. Lisanti , Cell Cycle 2009, 8, 3984 19923890 10.4161/cc.8.23.10238

[129] R. S. BelAiba , T. Djordjevic , S. Bonello , D. Flugel , J. Hess , T. Kietzmann , A. Gorlach , Biol. Chem. 2004, 385, 249 15134338 10.1515/BC.2004.019

[130] E. Kotake‐Nara , K. Saida , Neurosci. Lett. 2007, 422, 223 17629402 10.1016/j.neulet.2007.06.026

[131] C. Trimmer , F. Sotgia , D. Whitaker‐Menezes , R. M. Balliet , G. Eaton , U. E. Martinez‐Outschoorn , S. Pavlides , A. Howell , R. V. Iozzo , R. G. Pestell , P. E. Scherer , F. Capozza , M. P. Lisanti , Cancer Biol. Ther. 2011, 11, 383 21150282 10.4161/cbt.11.4.14101 PMC3047109

[132] T. Fiaschi , A. Marini , E. Giannoni , M. L. Taddei , P. Gandellini , A. De Donatis , M. Lanciotti , S. Serni , P. Cirri , P. Chiarugi , Cancer Res. 2012, 72, 5130 22850421 10.1158/0008-5472.CAN-12-1949

[133] M. S. Ullah , A. J. Davies , A. P. Halestrap , J. Biol. Chem. 2006, 281, 9030 16452478 10.1074/jbc.M511397200

[134] D. Whitaker‐Menezes , U. E. Martinez‐Outschoorn , Z. Lin , A. Ertel , N. Flomenberg , A. K. Witkiewicz , R. C. Birbe , A. Howell , S. Pavlides , R. Gandara , R. G. Pestell , F. Sotgia , N. J. Philp , M. P. Lisanti , Cell Cycle 2011, 10, 1772 21558814 10.4161/cc.10.11.15659 PMC3142461

[135] C. M. Sousa , D. E. Biancur , X. Wang , C. J. Halbrook , M. H. Sherman , L. Zhang , D. Kremer , R. F. Hwang , A. K. Witkiewicz , H. Ying , J. M. Asara , R. M. Evans , L. C. Cantley , C. A. Lyssiotis , A. C. Kimmelman , Nature 2016, 536, 479 27509858 10.1038/nature19084 PMC5228623

[136] L. Yang , A. Achreja , T. L. Yeung , L. S. Mangala , D. Jiang , C. Han , J. Baddour , J. C. Marini , J. Ni , R. Nakahara , S. Wahlig , L. Chiba , S. H. Kim , J. Morse , S. Pradeep , A. S. Nagaraja , M. Haemmerle , N. Kyunghee , M. Derichsweiler , T. Plackemeier , I. Mercado‐Uribe , G. Lopez‐Berestein , T. Moss , P. T. Ram , J. Liu , X. Lu , S. C. Mok , A. K. Sood , D. Nagrath , Cell metabolism 2016, 24, 685 27829138 10.1016/j.cmet.2016.10.011 PMC7329194

[137] H. Zhao , L. Yang , J. Baddour , A. Achreja , V. Bernard , T. Moss , J. C. Marini , T. Tudawe , E. G. Seviour , F. A. San Lucas , H. Alvarez , S. Gupta , S. N. Maiti , L. Cooper , D. Peehl , P. T. Ram , A. Maitra , D. Nagrath , Elife 2016, 5, e10250 26920219 10.7554/eLife.10250 PMC4841778

[138] S. X. Chen , X. E. Xu , X. Q. Wang , S. J. Cui , L. L. Xu , Y. H. Jiang , Y. Zhang , H. B. Yan , Q. Zhang , J. Qiao , P. Y. Yang , F. Liu , J. Proteomics 2014, 110, 155 25118038 10.1016/j.jprot.2014.07.031

[139] A. Bronisz , J. Godlewski , J. A. Wallace , A. S. Merchant , M. O. Nowicki , H. Mathsyaraja , R. Srinivasan , A. J. Trimboli , C. K. Martin , F. Li , L. Yu , S. A. Fernandez , T. Pecot , T. J. Rosol , S. Cory , M. Hallett , M. Park , M. G. Piper , C. B. Marsh , L. D. Yee , R. E. Jimenez , G. Nuovo , S. E. Lawler , E. A. Chiocca , G. Leone , M. C. Ostrowski , Nat. Cell Biol. 2011, 14, 159 22179046 10.1038/ncb2396 PMC3271169

[140] A. De Boeck , A. Hendrix , D. Maynard , M. Van Bockstal , A. Daniels , P. Pauwels , C. Gespach , M. Bracke , O. De Wever , Proteomics 2013, 13, 379 23175172 10.1002/pmic.201200179

[141] E. Bagordakis , I. Sawazaki‐Calone , C. C. Macedo , C. M. Carnielli , C. E. de Oliveira , P. C. Rodrigues , A. L. Rangel , J. N. Dos Santos , J. Risteli , E. Graner , T. Salo , A. F. Paes Leme , R. D. Coletta , Tumour Biol. 2016, 37, 9045 26762409 10.1007/s13277-015-4629-y

[142] M. M. Koczorowska , S. Tholen , F. Bucher , L. Lutz , J. N. Kizhakkedathu , O. De Wever , U. F. Wellner , M. L. Biniossek , A. Stahl , S. Lassmann , O. Schilling , Mol. Oncol. 2016, 10, 40 26304112 10.1016/j.molonc.2015.08.001 PMC5528924

[143] A. M. Santos , J. Jung , N. Aziz , J. L. Kissil , E. Pure , J. Clin. Invest. 2009, 119, 3613 19920354 10.1172/JCI38988 PMC2786791

[144] C. Holmberg , B. Ghesquiere , F. Impens , K. Gevaert , J. D. Kumar , N. Cash , S. Kandola , P. Hegyi , T. C. Wang , G. J. Dockray , A. Varro , J. Proteome Res. 2013, 12, 3413 23705892 10.1021/pr400270q PMC3709265

[145] M. Shimoda , S. Principe , H. W. Jackson , V. Luga , H. Fang , S. D. Molyneux , Y. W. Shao , A. Aiken , P. D. Waterhouse , C. Karamboulas , F. M. Hess , T. Ohtsuka , Y. Okada , L. Ailles , A. Ludwig , J. L. Wrana , T. Kislinger , R. Khokha , Nat. Cell Biol. 2014, 16, 889. 25150980 10.1038/ncb3021

[146] A. Byron , J. D. Humphries , M. J. Humphries , Int. J. Exp. Pathol. 2013, 94, 75 23419153 10.1111/iep.12011 PMC3607136

[147] S. T. Rashid , J. D. Humphries , A. Byron , A. Dhar , J. A. Askari , J. N. Selley , D. Knight , R. D. Goldin , M. Thursz , M. J. Humphries , J. Proteome Res. 2012, 11, 4052 22694338 10.1021/pr3000927 PMC3411196

[148] A. Naba , K. R. Clauser , D. R. Mani , S. A. Carr , R. O. Hynes , Sci. Rep. 2017, 7, 40495 28071719 10.1038/srep40495 PMC5223159

[149] A. Naba , O. M. T. Pearce , A. Del Rosario , D. Ma , H. Ding , V. Rajeeve , P. R. Cutillas , F. R. Balkwill , R. O. Hynes , J. Proteome Res. 2017, 16, 3083 28675934 10.1021/acs.jproteome.7b00191 PMC8078728

[150] A. E. Mayorca‐Guiliani , C. D. Madsen , T. R. Cox , E. R. Horton , F. A. Venning , J. T. Erler , Nat. Med. 2017, 23, 890 28604702 10.1038/nm.4352

[151] A. Naba , K. R. Clauser , S. Hoersch , H. Liu , S. A. Carr , R. O. Hynes , Mol. Cell. Proteomics 2012, 11, M111 014647 22159717 10.1074/mcp.M111.014647 PMC3322572

[152] D. Szklarczyk , A. Franceschini , S. Wyder , K. Forslund , D. Heller , J. Huerta‐Cepas , M. Simonovic , A. Roth , A. Santos , K. P. Tsafou , M. Kuhn , P. Bork , L. J. Jensen , C. von Mering , Nucleic Acids Res. 2015, 43, D447 25352553 10.1093/nar/gku1003 PMC4383874

[153] U.. Consortium , Nucleic Acids Res. 2010, 38, D142 19843607

[154] A. Naba , K. R. Clauser , H. Ding , C. A. Whittaker , S. A. Carr , R. O. Hynes , Matrix Biol. 2016, 49, 10 26163349 10.1016/j.matbio.2015.06.003 PMC5013529

[155] M. S. Cline , M. Smoot , E. Cerami , A. Kuchinsky , N. Landys , C. Workman , R. Christmas , I. Avila‐Campilo , M. Creech , B. Gross , K. Hanspers , R. Isserlin , R. Kelley , S. Killcoyne , S. Lotia , S. Maere , J. Morris , K. Ono , V. Pavlovic , A. R. Pico , A. Vailaya , P. L. Wang , A. Adler , B. R. Conklin , L. Hood , M. Kuiper , C. Sander , I. Schmulevich , B. Schwikowski , G. J. Warner , T. Ideker , G. D. Bader , Nat. Protoc. 2007, 2, 2366 17947979 10.1038/nprot.2007.324 PMC3685583

[156] R. Aebersold , M. Mann , Nature 2016, 537, 347 27629641 10.1038/nature19949

[157] A. I. Lamond , M. Uhlen , S. Horning , A. Makarov , C. V. Robinson , L. Serrano , F. U. Hartl , W. Baumeister , A. K. Werenskiold , J. S. Andersen , O. Vorm , M. Linial , R. Aebersold , M. Mann , Mol. Cell. Proteomics 2012, 11, O112 017731 22311636 10.1074/mcp.O112.017731 PMC3316737

[158] R. A. Scheltema , J. P. Hauschild , O. Lange , D. Hornburg , E. Denisov , E. Damoc , A. Kuehn , A. Makarov , M. Mann , Mol. Cell. Proteomics 2014, 13, 3698 25360005 10.1074/mcp.M114.043489 PMC4256516

[159] F. Yang , Y. Shen , D. G. Camp, 2nd , R. D. Smith , Expert Rev. Proteomics 2012, 9, 129 22462785 10.1586/epr.12.15 PMC3337716

[160] N. A. Kulak , P. E. Geyer , M. Mann , Mol. Cell. Proteomics 2017, 16, 694 28126900 10.1074/mcp.O116.065136 PMC5383787

[161] D. B. Bekker‐Jensen , C. D. Kelstrup , T. S. Batth , S. C. Larsen , C. Haldrup , J. B. Bramsen , K. D. Sorensen , S. Hoyer , T. F. Orntoft , C. L. Andersen , M. L. Nielsen , J. V. Olsen , Cell Syst. 2017, 4, 587–599 e584 10.1016/j.cels.2017.05.009 PMC5493283 28601559

[162] J. Cox , M. Mann , Nat. Biotechnol. 2008, 26, 1367 19029910 10.1038/nbt.1511

[163] H. L. Rost , Y. Liu , G. D'Agostino , M. Zanella , P. Navarro , G. Rosenberger , B. C. Collins , L. Gillet , G. Testa , L. Malmstrom , R. Aebersold , Nat. Methods 2016, 13, 777 27479329 10.1038/nmeth.3954 PMC5008461

[164] S. E. Ong , B. Blagoev , I. Kratchmarova , D. B. Kristensen , H. Steen , A. Pandey , M. Mann , Mol. Cell. Proteomics 2002, 1, 376 12118079 10.1074/mcp.m200025-mcp200

[165] P. L. Ross , Y. N. Huang , J. N. Marchese , B. Williamson , K. Parker , S. Hattan , N. Khainovski , S. Pillai , S. Dey , S. Daniels , S. Purkayastha , P. Juhasz , S. Martin , M. Bartlet‐Jones , F. He , A. Jacobson , D. J. Pappin , Mol. Cell. Proteomics 2004, 3, 1154 15385600 10.1074/mcp.M400129-MCP200

[166] N. P. Gauthier , B. Soufi , W. E. Walkowicz , V. A. Pedicord , K. J. Mavrakis , B. Macek , D. Y. Gin , C. Sander , M. L. Miller , Nat. Methods 2013, 10, 768 23817070 10.1038/nmeth.2529 PMC4002004

[167] C. J. Tape , I. C. Norrie , J. D. Worboys , L. Lim , D. A. Lauffenburger , C. Jorgensen , Mol. Cell. Proteomics 2014, 13, 1866 24820872 10.1074/mcp.O113.037119 PMC4083121

[168] C. J. Tape , S. Ling , M. Dimitriadi , K. M. McMahon , J. D. Worboys , H. S. Leong , I. C. Norrie , C. J. Miller , G. Poulogiannis , D. A. Lauffenburger , C. Jorgensen , Cell 2016, 165, 1818 27315484 10.1016/j.cell.2016.05.079 PMC5628167

[169] A. Santi , A. Caselli , F. Ranaldi , P. Paoli , C. Mugnaioni , E. Michelucci , P. Cirri , Biochim. Biophys. Acta 2015, 1853, 3211 26384873 10.1016/j.bbamcr.2015.09.013

[170] O. Rechavi , M. Kalman , Y. Fang , H. Vernitsky , J. Jacob‐Hirsch , L. J. Foster , Y. Kloog , I. Goldstein , Nat. Methods 2010, 7, 923 20935649 10.1038/nmeth.1513

[171] J. Albrengues , T. Bertero , E. Grasset , S. Bonan , M. Maiel , I. Bourget , C. Philippe , C. Herraiz Serrano , S. Benamar , O. Croce , V. Sanz‐Moreno , G. Meneguzzi , C. C. Feral , G. Cristofari , C. Gaggioli , Nat. Commun. 2015, 6, 10204. 26667266 10.1038/ncomms10204 PMC4682161

[172] D. Zhang , Y. Wang , Z. Shi , J. Liu , P. Sun , X. Hou , J. Zhang , S. Zhao , B. P. Zhou , J. Mi , Cell Rep. 2015, 10, 1335 25732824 10.1016/j.celrep.2015.02.006

[173] E. McDonnell , S. B. Crown , D. B. Fox , B. Kitir , O. R. Ilkayeva , C. A. Olsen , P. A. Grimsrud , M. D. Hirschey , Cell Rep. 2016, 17, 1463 27806287 10.1016/j.celrep.2016.10.012 PMC5123807

[174] D. R. Bandura , V. I. Baranov , O. I. Ornatsky , A. Antonov , R. Kinach , X. Lou , S. Pavlov , S. Vorobiev , J. E. Dick , S. D. Tanner , Anal. Chem. 2009, 81, 6813 19601617 10.1021/ac901049w

[175] S. C. Bendall , E. F. Simonds , P. Qiu , A. D. Amir el , P. O. Krutzik , R. Finck , R. V. Bruggner , R. Melamed , A. Trejo , O. I. Ornatsky , R. S. Balderas , S. K. Plevritis , K. Sachs , D. Pe'er , S. D. Tanner , G. P. Nolan , Science 2011, 332, 687 21551058 10.1126/science.1198704 PMC3273988

[176] S. Chevrier , J. H. Levine , V. R. T. Zanotelli , K. Silina , D. Schulz , M. Bacac , C. H. Ries , L. Ailles , M. A. S. Jewett , H. Moch , M. van den Broek , C. Beisel , M. B. Stadler , C. Gedye , B. Reis , D. Pe'er , B. Bodenmiller , Cell 2017, 169, 736‐749 e718 10.1016/j.cell.2017.04.016 PMC5422211 28475899

[177] P. Chaurand , S. A. Schwartz , R. M. Caprioli , Curr. Opin. Chem. Biol. 2002, 6, 676 12413553 10.1016/s1367-5931(02)00370-8

[178] P. J. Roach , J. Laskin , A. Laskin , Analyst 2010, 135, 2233 20593081 10.1039/c0an00312c

[179] C. C. Hsu , P. T. Chou , R. N. Zare , Anal. Chem. 2015, 87, 11171 26509582 10.1021/acs.analchem.5b03389

